# Murine Esophagus Expresses Glial-Derived Central Nervous System Antigens

**DOI:** 10.3390/ijms22063233

**Published:** 2021-03-22

**Authors:** Christopher Kapitza, Rittika Chunder, Anja Scheller, Katherine S. Given, Wendy B. Macklin, Michael Enders, Stefanie Kuerten, Winfried L. Neuhuber, Jürgen Wörl

**Affiliations:** 1 Institute of Anatomy and Cell Biology, Friedrich-Alexander-Universität Erlangen-Nürnberg (FAU), 91054 Erlangen, Germany; christopher.kapitza@fau.de (C.K.); rittika.chunder@fau.de (R.C.); michi.enders@fau.de (M.E.); stefanie.kuerten@fau.de (S.K.); winfried.neuhuber@fau.de (W.L.N.); 2 University of Saarland, Department of Molecular Physiology, Center for Integrative Physiology and Molecular Medicine (CIPMM), 66421 Homburg, Germany; anja.scheller@uks.eu; 3 Department of Cell and Developmental Biology, University of Colorado School of Medicine, Aurora, CO 80045, USA; katherine.given@ucdenver.edu (K.S.G.); wendy.macklin@ucdenver.edu (W.B.M.); 4 Department of Neuroanatomy, Institute of Anatomy, University Hospitals Bonn, University Bonn, 53115 Bonn, Germany

**Keywords:** autoantibodies, enteric glia, enteric nervous system, esophagus, dysphagia, glial fibrillary acidic protein, myelin basic protein, motor endplate, multiple sclerosis, proteolipid protein

## Abstract

Multiple sclerosis (MS) has been considered to specifically affect the central nervous system (CNS) for a long time. As autonomic dysfunction including dysphagia can occur as accompanying phenomena in patients, the enteric nervous system has been attracting increasing attention over the past years. The aim of this study was to identify glial and myelin markers as potential target structures for autoimmune processes in the esophagus. RT-PCR analysis revealed glial fibrillary acidic protein (GFAP), proteolipid protein (PLP), and myelin basic protein (MBP) expression, but an absence of myelin oligodendrocyte glycoprotein (MOG) in the murine esophagus. Selected immunohistochemistry for GFAP, PLP, and MBP including transgenic mice with cell-type specific expression of PLP and GFAP supported these results by detection of (1) GFAP, PLP, and MBP in Schwann cells in skeletal muscle and esophagus; (2) GFAP, PLP, but no MBP in perisynaptic Schwann cells of skeletal and esophageal motor endplates; (3) GFAP and PLP, but no MBP in glial cells surrounding esophageal myenteric neurons; and (4) PLP, but no GFAP and MBP in enteric glial cells forming a network in the esophagus. Our results pave the way for further investigations regarding the involvement of esophageal glial cells in the pathogenesis of dysphagia in MS.

## 1. Introduction

Multiple sclerosis (MS) is a chronic inflammatory, neurodegenerative disease of the central nervous system (CNS) causing myelin sheath destruction. Due to the neuronal damage, the signal transmission in the CNS gets disrupted [1,2]. As a consequence, patients typically suffer from a broad variety of symptoms like fatigue, sensory disturbances, paresthesia, chronical pain, and spasticity to paralysis, depending on the affected areas [3].

Historically, the CNS has been considered to be the primary target of autoimmunity in MS with evidence for antibody-dependent pathomechanisms against CNS myelin- and glial-derived antigens in a group of patients [4]. However, more recent studies have discussed the role of the enteric nervous system (ENS) as a potential target for autoimmunity [5,6], hence challenging our current understanding of the immunopathogenesis of MS. For instance, dysregulated gastrointestinal functions, anorectal dysfunction, and fecal incontinence in MS patients are examples that may include disruptions in the regulation of the ENS [7,8,9].

An underestimated problem—affecting more than a third of all MS patients and more than 65% of advanced cases—is difficulty in swallowing and esophageal dysphagia [10,11]. While difficulties in swallowing may already develop in patients with mild impairment, it occurs more frequently in MS patients with severe disability, suggesting a direct correlation between disease severity and dysphagia [12]. Swallowing problems not only result in reduced life quality but esophageal dysphagia also accounts for a high risk factor for aspiration-associated pneumonia, a common cause for morbidity and increased mortality in the progressive stage of MS [10,13].

Dysphagia can result from a combination of several factors including involvement of the tractus corticonuclearis in the brainstem, cerebellar dysfunction as well as lesions of the lower cranial nerves [10]. While most of the MS research focuses on pathological changes within the CNS [14,15], only a limited number of studies is addressing the involvement of the esophagus in the pathophysiology of MS-related dysphagia [11,16]. Furthermore, dysphagia may also result from damaged and subsequently lost ENS function [17] with studies supporting the hypothesis that patients with esophageal dysfunction harbor anti-enteric neuronal antibodies in their sera [18].

The innervation of the esophagus is complex. Striated muscle is supplied by cholinergic vagal motor neurons in the brainstem nucleus ambiguous and nitrergic/peptidergic neurons in enteric ganglia [19,20,21,22]. In addition, there is sensory innervation from the nodose and dorsal root ganglia. Thus, a variety of glial cells, both myelinating and non-myelinating as well as enteric, was to be expected and may represent potential targets of immune-mediated damage in MS patients. In this context, the antigenic characterization of the glial cells in the esophagus remains as an important step to understand if and how the esophagus is affected and undergoes pathological changes in this disease.

While there are studies primarily focusing on the characterization of enteric neurons in the esophagus [21,22,23,24], there are only a few studies available on the expression of glial-derived antigens in this organ [25,26]. To address this gap in knowledge, in the present study, we have first characterized the esophagus on a mRNA level applying a pre-selected panel of common glial markers, followed by their expression in immunohistochemical stainings of healthy C57BL/6J mouse tissue. In addition, we used mono- and double transgenic mouse lines for proteolipid protein (PLP) and glial fibrillary acidic protein (GFAP) to support our immunohistochemical investigations. The tibialis anterior muscle was included in order to compare glial cells associated with motor innervation of striated esophageal muscle with those in a representative skeletal muscle.

## 2. Results

### 2.1. The Esophagus Expresses CNS Typical Markers at the mRNA-Level

For antigenic characterization of the esophagus, six C57BL/6J mice (*n* = 3 female, *n* = 3 male) were used to compare the esophagus with the other regions of the ENS, with colon ascendens and jejunum included as controls. Furthermore, the musculus tibialis anterior was used as a skeletal muscle reference for the esophagus. On the other hand, different regions of the brain (including the cerebrum, cerebellum, and brain stem) served as controls for CNS antigens. Since results from individual mice did not differ, data were pooled for expression analysis.

We screened for expression of PLP, myelin basic protein (MBP), myelin associated glycoprotein (MAG), myelin oligodendrocyte glycoprotein (MOG), GFAP, and oligodendrocyte-specific protein (OSP, also known as claudin-11). PLP and MBP are the two most abundant myelin proteins of the myelin sheath and their importance for inducing experimental autoimmune encephalomyelitis (EAE), one mouse model for MS, has been previously discussed [27,28,29]. Furthermore, PLP in combination with GFAP identifies a unique population of glial cells in the ENS of the lower gastrointestinal tract [30,31]. MOG, which is predominantly expressed in CNS myelin, is a potential target for cellular and humoral immune response in EAE and MS [32]. MAG is a common myelin antigen that is expressed both in the CNS and the PNS [33] and is also an antigenic target in peripheral neuropathies [34,35].

**Figure 1:**
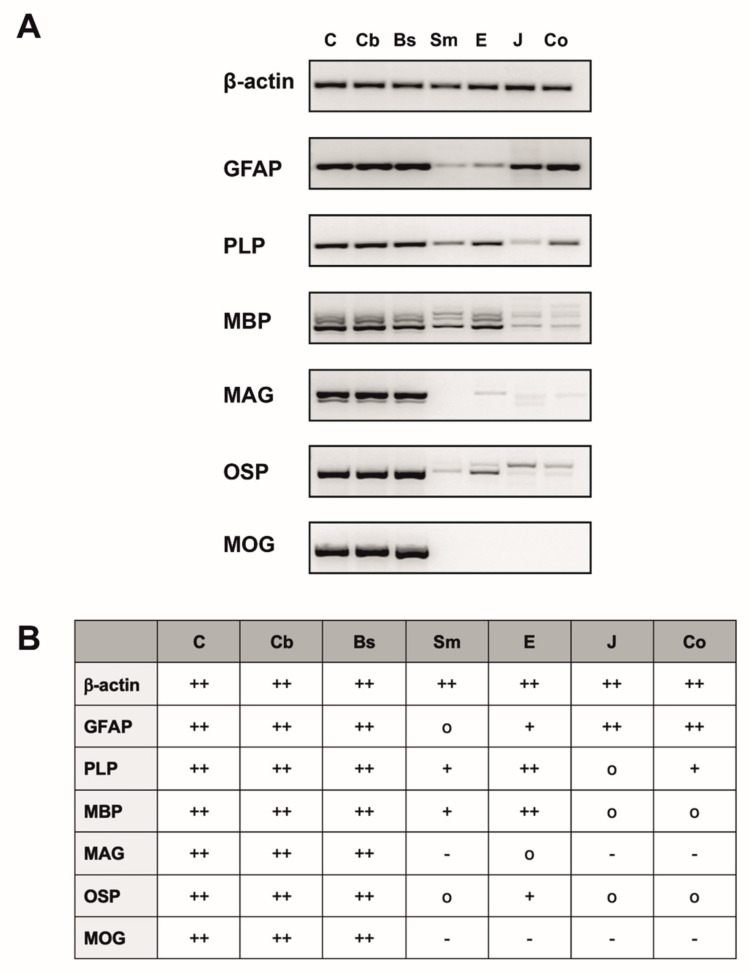
RT-PCR analysis for β-actin, glial fibrillary acidic protein (GFAP), proteolipid protein (PLP), myelin basic protein (MBP), myelin associated glycoprotein (MAG), oligodendrocyte-specific protein (OSP) and myelin oligodendrocyte glycoprotein (MOG) of *n* = 6 mice. (**A**) Overview of different expression levels of the examined markers with β-actin used as housekeeping gene, shown by one representative mouse. (**B**) Table summarizing the PCR results of six mice and showing the expression profile of the different tissues investigated with + indicating a “high expression”, + indicating a “medium–low expression”, o indicating a “very low expression” and–indicating no expression. Since results from individual mice did not differ, data were pooled. C: Cerebrum; Cb: Cerebellum; Bs: Brain stem; Sm: Skeletal muscle (M. tibialis anterior); E: Esophagus; J: Jejunum; Co: Colon ascendens; GFAP: Glial fibrillary acidic protein; PLP: Proteolipid protein; MBP: Myelin basic protein; MAG: Myelin-associated glycoprotein; OSP: Oligodendrocyte-specific protein; MOG: Myelin oligodendrocyte glycoprotein.

Different expression levels of the investigated glial antigens were detected at the mRNA level in the esophagus under physiological conditions. mRNA expression of PLP and MBP were the highest in comparison to low levels of GFAP and OSP expression (Figure 1. MAG mRNA transcripts were detected at a very low level. As expected, MOG, which is considered to be a CNS specific myelin marker, was not expressed in the esophagus. We also found the same expression of glial and myelin markers in skeletal muscle, jejunum and colon. The only differences were (1) the expression of MAG in the esophagus, which was completely absent in skeletal muscle, jejunum and colon; (2) the different expression levels in all investigated non-CNS tissues. Furthermore, the different regions of CNS tissues, that were chosen as positive controls, all showed a high expression of all investigated markers. Based on these results, we decided to prove the presence of MBP, PLP and GFAP in the esophagus by immunohistochemistry in a further step.

### 2.2. Distribution of MBP, PLP and GFAP in the Cerebellum

Cryosections of cerebellar tissue were chosen as positive controls for MBP, PLP, and GFAP immunohistochemistry. For a simple differentiation between the three main laminae of the cerebellar cortex, (1) stratum moleculare, (2) stratum purkinjense, and (3) stratum granulare (Supplementary Figure S1A), calbindin D28k (CALB) was chosen as a marker. This calcium binding protein was found in Purkinje cells and therefore stained the prominent, pyknic cell bodies located in the stratum purkinjense as well as their ramification in the stratum moleculare, caused by the protruding dendrites. The third lamina, the stratum granulare, which is directly adjacent to the medullary layer, however, showed only little positiveness for CALB but in contrast numerous granule cells, indicated with Hoechst nuclear staining (Supplementary Figure S1A).

In order to identify the distribution and the co-localization of PLP and MBP, we combined these two myelin markers with CALB and Hoechst in a quadruple staining (N° ①, Table 1; Supplementary Figure S1A,B). PLP and MBP likewise indicated axons myelinated by oligodendrocytes in the stratum granulare and the white matter. Moreover, some PLP^+^- and MBP^+^ -fibers reached the stratum moleculare ensheathing CALB^+^-afferences, which most likely represent afferences from the inferior olive [36]. As expected, in all cases, MBP and PLP appeared completely congruent due to their appearance in the myelin sheath (Supplementary Figure S1C–E). To investigate differences in the organization of GFAP^+^ -glial cells and myelinating glial cells, we performed another quadruple staining (N° ②, Table 1; Supplementary Figure S1F–G), combining PLP, GFAP, CALB, and Hoechst. In contrast to the distribution for PLP as described above, GFAP^+^-cells occurred widely spread in all different segments of the cerebellum and appeared to not be highly co-located with PLP^+^ -glial cells (Supplementary Figure S1H–J). In the stratum granulare, the longish, cross-linked protrusions of Bergmann Glia, unipolar astrocyte-like cells, could be found, leading to a network surrounding the Purkinje cells (Supplementary Figure S1I) [37]. Throughout the stratum moleculare a high number of prolate, radial aligned fibers were detectable, which then terminated at the pial surface, forming the membrana limitans gliae superficialis (Supplementary Figure S1G). Taken together, these results prove the specificity of the used antibodies.

### 2.3. Glial Cells in Neuromuscular Junctions of the Tibialis Anterior Muscle

To address the question, how the three main glial markers of this study are involved in the formation of the neuromuscular junction (NMJ) in a skeletal muscle, which serves as a comparison control for the striated esophageal muscle, cryosections of the musculus tibialis anterior were applied for immunohistochemical triple stainings (Supplementary Figure S2A–P). Each time, synaptophysin as a marker for cholinergic vesicles, and α-Bungarotoxin (α-BT), as a specific marker for motor endplates binding to postsynaptic acetylcholine receptors, were used, and showed a linked distribution in the endplate region: Synaptophysin as a presynaptic marker was surrounded by the contour of α-BT, which traced the postsynaptic side of NMJ. In general, synaptophysin was abundant in the endplate region, while its expression along the axon decreased proximally with distance to the endplate (Supplementary Figure S2D,H,L).

Furthermore, GFAP (N° ③, Table 1; Supplementary Figure S2A–D) could be found in myelinating glial cells of nerve fibers, which implies that peripheral Schwann cells contain GFAP as a cytoskeleton component [38]. Along their way to the motor endplate, the caliber of the myelinated fibers decreased until the endplate was reached. At this point, glial cells changed from myelinating to non-myelinating GFAP positive perisynaptic Schwann cells (PSC), resulting in a frame-like structure that accompanied the motor axon, but was predominantly located in the presynaptic region (Supplementary Figure S2B). The assumption of myelin loss of glial cells close to the endplate region was confirmed by the staining results of MBP (N° ④, Table 1; Supplementary Figure S2E–H) and PLP (N° ⑤, Table 1; Supplementary Figure S2I–P): In both cases, the axon that was eventually contacting the endplate was ensheathed by a myelinating Schwann cell—but shortly before the endplate, the myelin sheath ended and from there on, an unmyelinated synaptophysin^+^ -axon continued to the endplate region (Supplementary Figure S2E,I). While PLP could be found in the myelin sheath in all cases, we furthermore noticed a heterogeneous distribution of PLP in the terminal endplate region: In most cases, a faint staining for PLP could be seen, suggesting that the PSC are double positive for GFAP and PLP (Supplementary Figure S2B,N). However, in some cases, this could not be detected (Supplementary Figure S2J).

In order to confirm the staining results of the presynaptic marker synaptophysin, we used the neuro-axonal marker βIII-tubulin allowing to trace axons over a long period. Therefore, we applied a triple staining of βIII-tubulin, MBP, and α-BT (N° ⑧, Table 1; Supplementary Figure S3A–L). As expected, βIII-tubulin and synaptophysin showed the same distribution in the area around the NMJ (cf. Supplementary Figures S2D,H,L,P and S3J). However, in addition, βIII-tubulin made it possible to examine axonal structures to their full extent (Supplementary Figure S3B,E,G,J). In combination with the MBP antibody, we therefore could establish a useful panel of markers for the evaluation of axonal myelination (Supplementary Figure S3A–F). Again, we found an abrupt loss of myelin of the contacting efference prior to the endplate, which proved the results of the synaptophysin stainings (cf. Supplementary Figures S2E and S3I).

Together, these results suggest that in tibialis anterior muscle subsequently after myelinating GFAP^+^/PLP^+^ -glial cells, abundant non-myelinating GFAP^+^/PLP^+^ -glial cells (PSC) accompanied the terminal motor axon as a frame-like structure. In contrast, MBP distribution in this muscle was restricted to the myelin sheaths, which disappeared shortly before the endplate area and could not be found in the endplate region.

### 2.4. Glial Cells in Neuromuscular Junctions of the Esophagus

To determine whether glial cells in the NMJ of the esophagus are interacting with the endplate similarly to the ones in the skeletal muscle, we performed multi label immunohistochemistry on cryosections and whole mounts.

**Figure 2:**
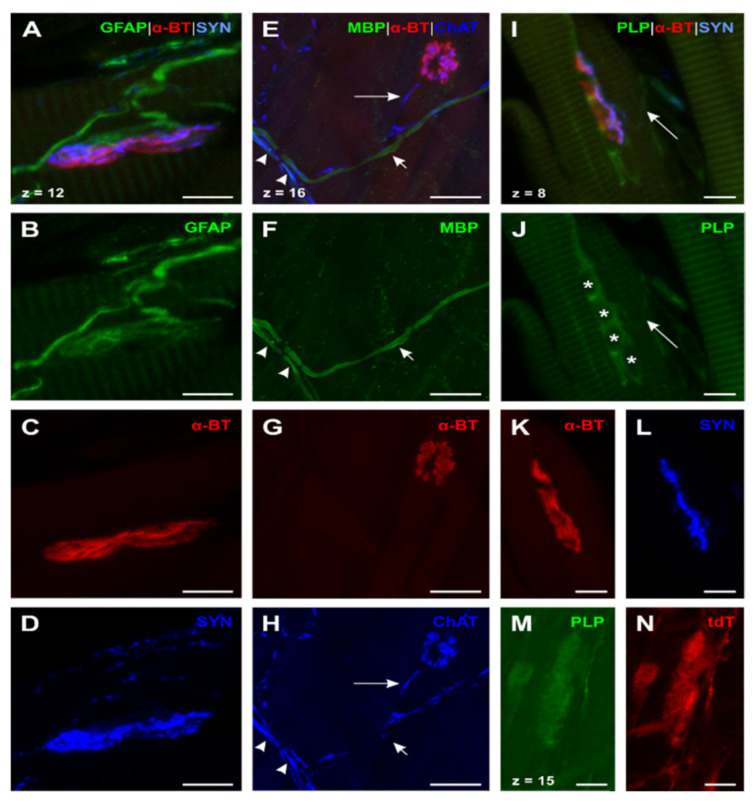
Expression of GFAP, MBP, and PLP in neuromuscular junction (NMJ) of the esophagus (**A**)–(**D**): Triple staining for GFAP, α-bungarotoxin (α-BT), and synaptophysin (SYN) (N° ③, Table 1) showed a similar distribution as the skeletal muscle with the exception that efferences were unmyelinated and had a smaller caliber. (**E**)–(**H**): Quadruple staining for MBP, α-BT, cholin acetyltransferase (ChAT), and Hoechst (Hoechst not shown; N° ①, Table 2). ChAT^+^-efferences which contact the motor endplate always lack myelin ((**E**) and (**H**), long arrow). Two further types of fibers could be detected: (1) Myelinated ChAT^+^-efferences ((**E**) and (**F**), arrowheads) and (2) myelinated ChAT^−^ -fibers ((**E**) and (**F**), short arrow)—the latter can be brought in line with esophageal afferences. (**I**) and (**L**): Quadruple staining for PLP, α-BT, SYN, and Hoechst (Hoechst not shown; N° ⑤; Table 1). In contrast to the skeletal muscle, PLP was present in all investigated NMJs as grouped PLP^+^ -glial cells around the endplate indicate (**I**) and (**J**). Nuclei of these PLP^+^ -PSCs are marked by asterisks (**J**); confirmed by Hoechst nuclear staining (not shown)). Contrary to the distribution of MBP, PLP could also be found throughout the Plexus myentericus as PLP^+^-efferences show ((**I**) and (**J**), long arrow). (**M**) and (**N**): Triple staining for Discosoma sp. red fluorescent protein (DsRed1), PLP, and protein gene product 9.5 (PGP 9.5) in PLP-CreERT2 x tdTomato (tdT) mice (anti-DsRed and PGP 9.5 not shown; N° ②, Table 2). The faint signal of PLP in the endplate region was confirmed by tdT expression as both PLP and tdT show the same distribution (**M**) and (**N**). α-BT: α-bungarotoxin; ChAT: Cholin acetyltransferase; DsRed: Discosoma sp. red fluorescent protein; GFAP: Glial fibrillary acidic protein; MBP: Myelin basic protein; PGP 9.5: Protein gene product 9.5; PLP: Proteolipid protein; SYN: Synaptophysin; tdT: tdTomato. Z-step = 1 µm (**A**)–(**D**); (**I**)–(**N**) and 0.8 µm (**E**)–(**H**); scale bars 10 µm (**A**)–(**D**), (**I**)–(**N**), 30 µm (**E**)–(**H**).

GFAP (N° ③, Table 1; Figure 2A–D) showed a similar distribution as in the skeletal muscle. However, motor axons were considerably smaller than in the skeletal muscle, supporting the impression that efferents in the esophagus always lack myelin before reaching the motor endplate. In addition, motor endplates appeared to be contacted by a more delicate web of GFAP^+^ -glial cells, surrounding the endplate region (Figure 2A,B).

**Figure 3:**
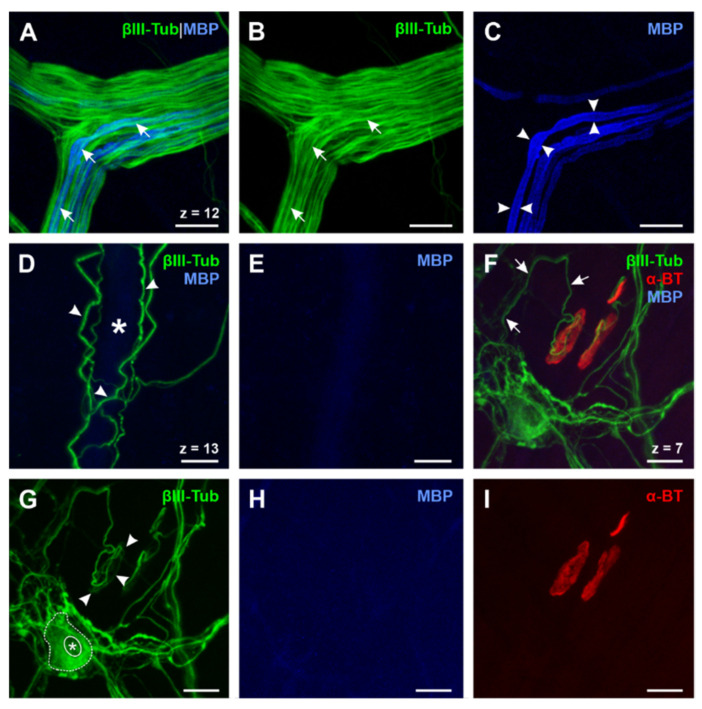
Expression of βIII-Tubulin, MBP, and α-BT in the esophagus (**A**)–(**E**): Triple whole mount staining of βIII-tubulin, MBP, and α-BT (α-BT not shown, N° ⑤, Table 2). Axons ((**A**) and (**B**), short arrows) of peripheral vagal nerve branches are only partially myelinated (**C**, arrowheads), while gracile blood vessel-related nerve fibers, which are tightly wrapped around the outer vessel wall, appear βIII-tubulin-positive (**D**, arrowheads) but always lack myelin since no MBP signal can be detected (**E**). The lumen of the blood vessel is marked by the asterisk (**D**). (**F**–**I**): Triple whole mount staining of βIII-tubulin, MBP and α-BT (N° ⑤, Table 2) reveals that endplate contacting efferent axons (**F**, short arrows) are always unmyelinated for a long distance (**H**) and form a framework in the presynaptic region of the NMJ (**G**, arrowheads). These findings confirm the results of the ChAT staining protocol (cf. Figure 2E–H). Moreover, βIII-tubulin can also be found in enteric neurons (**G**, dotted line; nucleus marked by asterisk) of the myenteric plexus and therefore proves to be a suitable neuro-axonal marker for the evaluation of the ENS in the esophagus. ChAT: Cholin acetyltransferase; MBP: Myelin basic protein; Z-step = 1 µm; scale bars 20 µm.

To verify the impression of an earlier myelin loss in comparison to skeletal muscle, we performed a quadruple whole mount staining of MBP, choline acetyltransferase (ChAT), α-BT, and Hoechst (Staining ①, Table 2; Figure 2E–H). We used the whole-mount approach to facilitate tracking of nerve fibers over a longer distance. These results demonstrate that efferent axons (identified as ChAT-positive) lose MBP long before they reach the endplate (Figure 2E, long arrow). Moreover, the majority of nerve fibers in the myenteric plexus was already unmyelinated with only a few myelinated nerve fibers present (Figure 2E,F). Some of the myelinated axons were ChAT^+^, thus identified as motor axons (Figure 2E, arrowheads). Others appeared to be negative for ChAT, assuming that these nerve fibers were afferents crossing the plexus on their way to the CNS (Figure 2E, short arrows). To demonstrate the specificity of the used ChAT antibody, we applied positive control experiments in M. tibialis anterior (Staining ⑥ and ⑦, Table 1, Supplementary Figure S4A–N). In a further triple whole mount staining of MBP, the neuro-axonal marker βIII-tubulin, and α-BT (Staining ⑤, Table 2) we could validate the results of the described ChAT-staining protocol as we found endplate contacting efferent axons in the esophagus unmyelinated (Figure 3F–I). Moreover, smaller peripheral branches of the vagal nerve contained only some myelinated nerve fibers while most of the axons lacked myelin (Figure 3A–C). In contrast to that, blood-vessel-related nerve fibers, which appeared wrapped around the outer vessel wall, always proved to be unmyelinated (Figure 3D–E).

In the next step, we set out to investigate whether there were differences in the distribution of MBP and PLP. Therefore, we at first used the same staining protocol for esophagus cryosections as for the skeletal muscle (Staining ⑤, Table 1; Figure 2I–L). The data show the same distribution pattern for PLP as for MBP. However, PLP was also present in the myenteric plexus and in the endplate region.

The oval shapes of the motor endplates, labeled by α-BT, were surrounded by grouped PLP^+^ -cells (Figure 2I,J). Unlike MBP, PLP was found throughout the myenteric plexus, as shown by PLP^+^ -axons terminating on motor endplates (Figure 2I,J, long arrow). Therefore, we assumed that PLP was on the one hand present in the myelin sheath of myelinating Schwann cells and on the other hand also in MBP^−^/PLP^+^-cells contacting the motor endplates. In order to confirm this hypothesis and to prove specific binding of the used PLP antibody, we established a staining protocol using whole mounts of transgenic tamoxifen-inducible PLP-CreERT2 x tdTomato mice. This transgenic mouse strain shows tdTomato (tdT) fluorescence in PLP^+^ -cells after tamoxifen induced recombination. Hence, we combined the PLP and anti-DsRed antibody staining—the latter reacting against tdT for signal amplification—with protein gene product 9.5 (PGP 9.5) staining (N° ②, Table 2). In this way, we could show a complete co-localization of the PLP antibody-staining and tdT (Figure 2M,N).

In summary, we were able to show that in the esophagus (1) GFAP and PLP were present in non-myelinating glial cells of axons, which terminated on esophageal motor endplates and in PSCs in motor endplates areas. (2) In agreement with the results in the tibialis anterior muscle, MBP was absent from endplate areas in the esophagus, but in contrast to the skeletal muscle, MBP was not present in Schwann cells of motor axons already long before they reached the motor endplate area.

### 2.5. Transgenic Mice Reveal the Presence of Different Types of Enteric Glial Cells in the Esophagus

**Figure 4:**
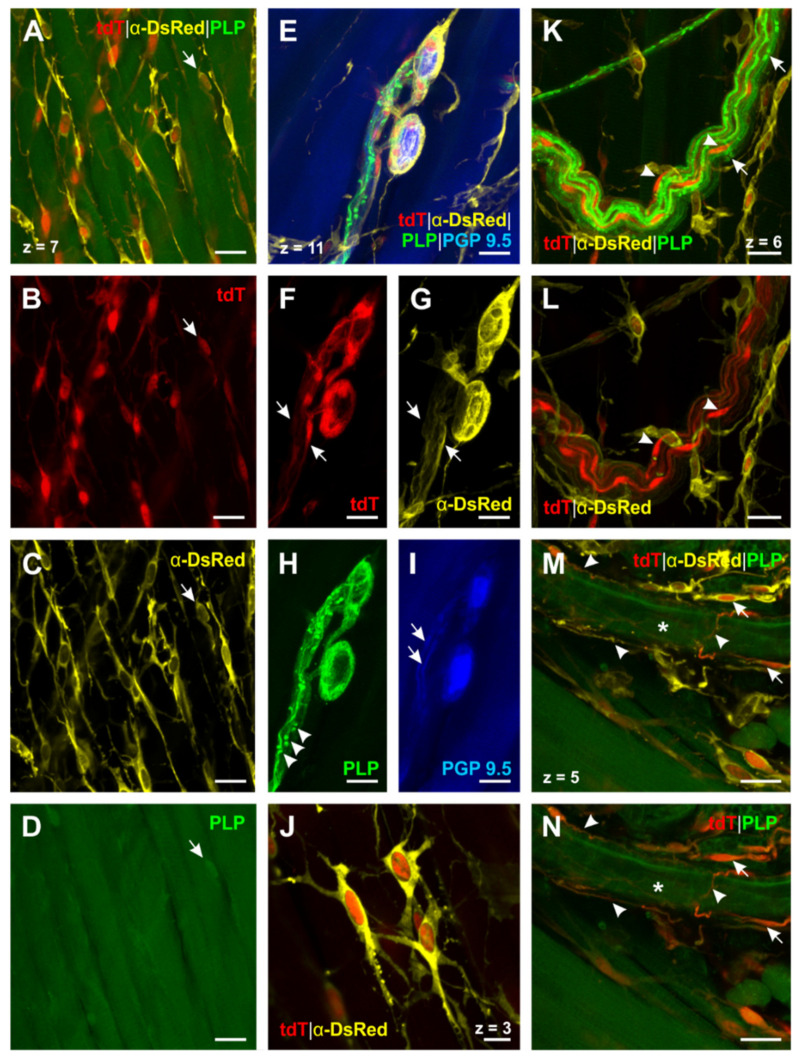
PLP^+^ -glial cells in the esophagus identified by triple staining for DsRed, PLP and PGP 9.5 in PLP-CreERT2 x tdT mice (N° ②, Table 2). (**A**)–(**D**) (PGP 9.5 not shown): PLP^+^ -glial cells form a meshwork throughout the esophagus. Some of these cells, located in the tunica muscularis, are arranged in parallel with the muscle fibers. While only a few of these cells can be detected by PLP antibody staining ((**A**–**D**), short arrow), transgenic PLP-CreERT2 x tdT mice reveal their distribution (cf. **A**–**C** vs. **D**). (**E**–**I**): PLP^+^ -glial cells interact very closely with enteric neurons as the processes of the EGCs are woven around the neurons (**E**–**H**). Interconnecting strands of enteric neurons (**I**, short arrows) are accompanied by PLP^+^ -glial cells (**F** and **G**, short arrows). PLP antibody staining indicates the contact zone of these cells (**H**, arrowheads). (**J**) (PGP 9.5 and PLP not shown): Close-up of PLP^+^ -EGCs; these cells are interconnected by their fine, filiform processes and therefore form a network of glial cells. (**K**–**L**) (PGP 9.5 not shown): PLP^+^ -myelin sheaths of a vagal nerve fiber bundle in the tunica adventitia can be identified by the PLP antibody (**K**, nodes of Ranvier are marked by short arrows). TdT expression shows the cell bodies of the peripheral myelinating Schwann cells ((**K** and **L**), arrowheads). (**M** and **N**) (PGP 9.5 not shown): Blood vessel-connected EGCs have a delicate morphology ((**M** and **N**), short arrows) and very long filiform processes, which appear woven around the outer vessel wall ((**M** and **N**), arrowheads). The lumen of the blood vessel is marked by the asterisk. DsRed: Discosoma sp. red fluorescent protein; PGP 9.5: protein gene product 9.5; PLP: proteolipid protein; tdT: tdTomato. Z-step = 1 µm; scale bars 20 µm (**A**–**I**, **K**–**N**), 10 µm (**J**).

**Figure 5:**
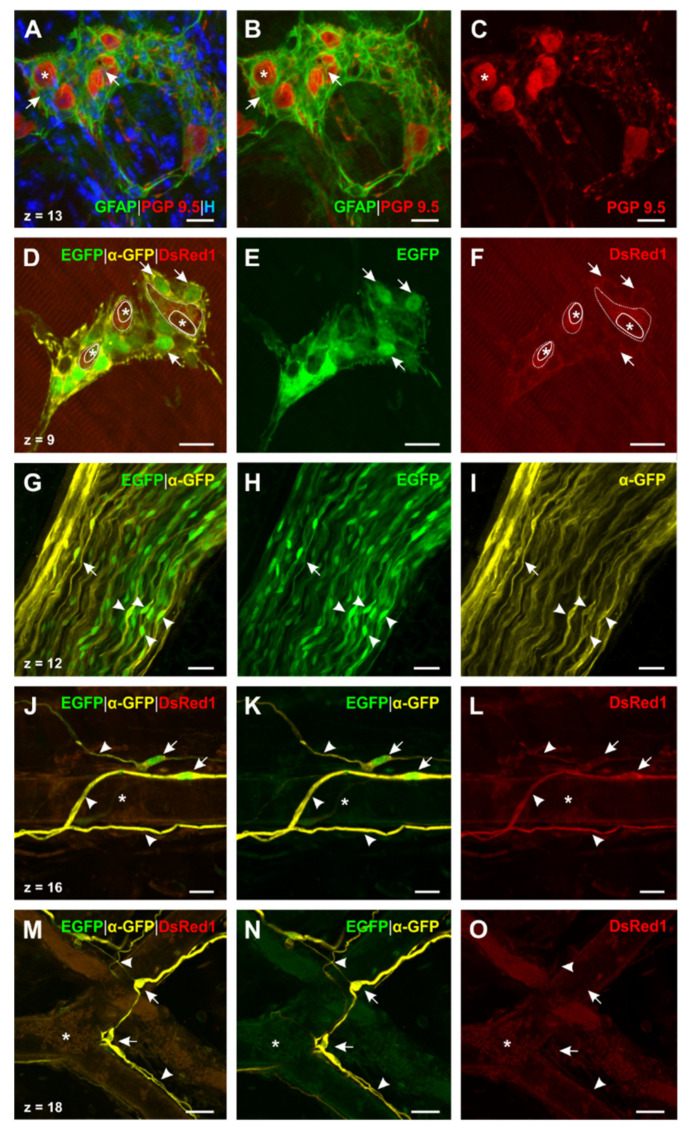
Distribution of PLP and GFAP in esophageal glial cells. (**A**–**C**): Triple staining for GFAP, PGP 9.5, and Hoechst (N° ③, Table 2) in wildtype mice shows the web, which is formed around the enteric ganglia of the myenteric plexus by GFAP^+^ -EGCs. Processes of these cells are woven around (**A** and **B**, short arrow) every neuron (**A**–**C**, asterisk). (**D**–**F**): Double staining for DsRed and GFP in GFAP-EGFP x PLP-DsRed1 mice (N° ④, Table 2; anti-DsRed not shown). Two different types of glial cells can be found: (1) GFAP^+^/PLP^+^ -glial cells, which appear most abundantly in myenteric ganglia (**D**–**F**, exemplarily indicated by short arrows). (2) GFAP^−^/PLP^+^ -glial cells, which can be only found directly surrounding the enteric neurons (**D** and **F**, dotted line; silhouettes of neurons are marked by asterisks). (**G**–**I**): Double staining for DsRed1 and GFP in GFAP-EGFP x PLP-DsRed1 mice (N° ④, Table 2; DsRed1 and anti-DsRed not shown). Longitudinal section of a vagal nerve fiber bundle of the tunica adventitia; GFAP can be detected in peripheral Schwann cells (arrowheads: cell bodies; short arrow: longish process). (**J**–**L**) and (**M**–**O:**) Double staining for DsRed1 and GFP in GFAP-EGFP x PLP-DsRed1 mice (N° ④, Table 2; anti-DsRed not shown). Detected blood vessel-connected EGCs show the same delicate morphology as the ones in PLP-CreERT2 x tdT mice (cf. Figure 3M,N). Remarkably, (1) GFAP^+^/PLP^+^-(**J**–**L**) and (2) GFAP^+^/PLP^−^ -glial cells (**M**–**O**) can be found as two different types of blood vessel connected glia. In all cases they show a similar distribution pattern, as their fine, long processes appear woven around (arrowheads) and the cell bodies closely located (short arrows) to the outer vessel wall. Lumina of the vessels are marked by asterisks. DsRed(1): Discosoma sp. red fluorescent protein (1); EGFP: Enhanced green fluorescent protein; GFAP: Glial fibrillary acidic protein; GFP: Green fluorescent protein; H: Hoechst; PGP 9.5: Protein gene product 9.5; PLP: Proteolipid protein; *Z*-step = 1 µm (**A**–**I**) and 0.5 µm (**J**–**O**); scale bars 20 µm (**A**–**F**), 25 µm (**G**–**O**).

To assess the distribution of PLP in glial cells of the esophageal ENS, we continued examining the whole mount stainings (N° ②, Table 2; Figure 4A–N) of PLP-CreERT2 x tdT mice. For a further comparison of GFAP and PLP we introduced a staining protocol for whole mounts of double transgenic GFAP-EGFP x PLP-DsRed1 mice (N° ③, Table 2; Figure 5D–O). In these animals, EGFP is driven by the human GFAP promoters and DsRed1 expression is controlled by the murine PLP promoter. In addition, we analyzed a triple staining for GFAP, PGP 9.5, and Hoechst (N° ④, Table 2; Figure 5A–C) in whole mounts of C57BL/6J mice.

General arrangement of glial cells. First, we scrutinized the distribution pattern of esophageal enteric glial cells (EGCs). We found a striking difference in their general arrangement: While GFAP^+^ -glial cells were mostly related to enteric ganglia and motor endplate contacting efferences, PLP^+^ -glial cells formed a meshwork of cells pervading the whole organ. Moreover, these glial cells were arranged in parallel with the muscle fibers of the tunica muscularis, giving the impression of enwrapping the muscle cells (Figure 4A–C). As we could not detect GFAP in these glial cells, the meshwork seemed to consist of single GFAP^−^/PLP^+^-cells, which were cross-linked by their processes. Due to their numerous finely branching protrusions, arising from their prominent round-oval soma, these cells showed a morphology reminiscent of astrocyte resembling glial cells (Figure 4J).

Enteric ganglia. We then focused on the morphology of glial cells surrounding enteric neurons in the esophagus. Through tdT expression in PLP-CreERT2 x tdT mice, PLP^+^ -glial cells could be detected around the enteric neurons (Figure 4F), even better distinguished by the DsRed antibody staining (Figure 4G). In addition, PLP^+^-glial cells also followed interconnecting strands of enteric neurons (Figure 4F,G, short arrows). The PLP antibody showed a similar distribution pattern, thus confirming the fluorescent protein results (Figure 4H). As a next step, we compared these findings in GFAP-EGFP x PLP-DsRed1 mice and GFAP whole mount stainings. In both cases, enteric ganglia were surrounded by GFAP^+^ -glial cells and embedded in a web formed by their filiform processes (Figure 5A,B). Moreover, the GFAP-EGFP x PLP-DsRed1 mice revealed the presence of at least two different cell types within the enteric ganglia: Most abundantly, GFAP^+^/ PLP^+^-glial cells were found, as the co-localization of EGFP–and DsRed1 indicated (Figure 5D–F). In addition, glial cells surrounding the enteric neurons appeared to be positive for PLP only (Figure 5D,F).

Glial cells of vagal nerve fibers. By screening whole mount preparations of the esophagus, we could also find bundles of vagal nerve fibers, adherent to the tunica adventitia. In PLP-CreERT2 x tdT mice oval cell bodies of PLP^+^ -glial cells, representing the peripheral Schwann cells, were present within the bundle as well as their gently stained cytoplasmatic protrusions. The PLP antibody staining showed the characteristic distribution of PLP in the myelin sheath around the axon (Figure 4K) and also allowed to identify ensheathing Schwann cells by nodes of Ranvier (Figure 4K). Moreover, it was noticed that the PLP antibody led to strong staining of the myelin sheath, whereas cell bodies of Schwann cells showed only little positivity in contrast to a strong cytosolic expression of tdT. We compared these results to the distribution of GFAP in GFAP-EGFP x PLP-DsRed1 mice (Figure 5G–I). We could see that cells with the same morphology as described above were also positive for GFAP, confirming that Schwann cells in the PNS contain GFAP as an intermediate filament.

Glial cells around blood vessels. Furthermore, we observed glial cells enwrapping blood vessels (Figure 4M,N,J–O). It was noticed that both GFAP^+^/PLP^+^-cells (Figure 5J–L) and GFAP^+^/PLP^−^-cells could be found (Figure 4M–O). Remarkably, vessel-related cells showed a delicate morphology: Close to the tunica adventitia of the blood vessels, their small, oval cell bodies could be found, emitting long, filiform protrusions of different calibers. These protrusions followed the course of the vessel in the tunica adventitia, forming glial networks.

In aggregate, we were able to demonstrate three different glial cell types in the esophagus using PLP and GFAP as a marker: GFAP^+^/PLP^−^ –, GFAP^+^/PLP^+^-, and GFAP^−^/PLP^+^-glial cells. Furthermore, the distribution of these cells in the esophagus showed some variability with GFAP^−^/PLP^+^-cells being the most abundant ones.

## 3. Discussion

This study represents the first detailed examination of different glial markers in the ENS of the murine esophagus, with special focus on GFAP, MBP, and PLP. As a screening method for glial cell marker expression, we used RT-PCR. In a further step, we applied immunohistochemical staining protocols in healthy C57BL/6J mice to show the presence of GFAP, MBP, and PLP at the protein level in the esophagus. In order to specify, confirm, and enlarge our findings, we finally completed our exploration by the use of mono- and double transgenic mouse lines for PLP and GFAP.

### 3.1. Presence of Glial Cell Specific Markers in the Esophageal Neuromuscular Junction

GFAP, known as a common glial marker, especially for astrocytes, was detected in the motor endplate region of the skeletal muscle. The delicate web formed by these glial cells can be reconciled with the morphology of perisynaptic Schwann cells [39]. While the expression of GFAP and PLP has already been described for NMJ in the skeletal muscle, the situation in the striated muscle of the esophagus is still not well understood [39,40]. Here we could confirm the findings in the skeletal muscle for the esophagus and demonstrate that esophageal PSC show a similar distribution of GFAP and PLP.

Although the function of these PSC is still not fully understood, there is evidence for their crucial role in axonal growth and regeneration as well as in the long-term maintenance of mature NMJ [39,41,42]. Moreover, due to their high expression of ion channels and neurotransmitter receptors they both resemble CNS astrocytes and take part in synaptic neurotransmission [43,44]. Therefore, they may interact in the so called tripartite synapse of the NMJ as the third partner, besides the presynaptic motor terminal and the postsynaptic muscle fiber [41].

### 3.2. Different Degrees of Myelination in the Myenteric Plexus

In our study, we were able to show that there are (1) myelinated (MBP^+^) ChAT^+^-fibers, (2) unmyelinated (MBP^−^) ChAT^+^-fibers, and (3) myelinated (MBP^+^) ChAT^−^ –fibers present in the myenteric plexus. ChAT is a well-known marker for motor efferents and hence allows to distinguish between motor efferents and afferents, which are both present in the myenteric plexus. While numerous unmyelinated efferent fibers were found, only few myelinated efferents were located in the myenteric plexus. In addition, in all cases, terminal motor axons, contacting the motor endplates, proved to be unmyelinated. These findings agree with results of previous studies [45,46], but the question concerning where the switch from myelinated to unmyelinated nerve fibers occurs remains still to be solved. One possible explanation could be that myelinated fibers lose their myelin sheath after entering the myenteric plexus [45,46]. Another possibility could be a lingering loss of myelin along the nerve fibers’ course, ending up in the unmyelinated, terminal axon accompanied by PSCs. Further investigations tracing myelinated nerve fibers on their way through the esophageal myenteric plexus are required in order to answer this question. However, the speed of signal transmission seems to be irrelevant in the terminal sections of efferents, as they are unmyelinated for long distances within the plexus.

As the third type of nerve fibers investigated in the myenteric plexus of the esophagus lacked ChAT but showed a myelin sheath, we came to the conclusion that they can be classified as afferent nerve fibers. Possible origins for these afferents are intraganglionic laminar endings (IGLEs) and intramuscular arrays (IMAs) [47,48,49], playing a crucial role in the sensory physiology of the esophagus: As far as is known, primary afferent fibers serve as receptors for (1) muscle tension, (2) mucosal mechanical and chemical stimuli, and (3) mucosal tension, and therefore are significant for swallowing. In this regard, several studies could confirm the correlation of sensory nerve impairment and functional disorders of the esophagus such as functional globus, noncardiac chest pain, and dysphagia [50,51,52].

### 3.3. Differentiation of Esophageal Enteric Glial Cells by the Use of Glial Markers

In the present study, we pursued the goal to characterize enteric glial cell populations of the esophagus. We could show that the common myelin marker PLP is widely expressed in the murine esophagus and, moreover, is noticeably more abundant than GFAP. As a consequence, we could identify three different glial cell types, depending on their expression of markers: GFAP^+^/PLP^−^, GFAP^+^/PLP^+^, and GFAP^−^/PLP^+^ -cells. These findings harmonize with the situation in the lower gastrointestinal tract [30].

Referring to morphology, Hanani and Reichenbach introduced a classification system of EGCs in the myenteric plexus of the guinea pig small intestine [53]. Based on microscopic investigations, they found four types of EGCs [53,54]: Type I cells are characterized by a star-shaped soma with short and irregularly branched processes, therefore also called ‘protoplasmic’. Type II (‘fibrous’) gliocytes appear as elongated EGCs within interganglionic fiber tracts. Type III (‘mucosal’) glial cells show numerous long-branched protrusions and can be found in subepithelial areas. ‘Intramuscular’ glial cells, representing type IV–gliocytes, feature a long-shaped cell body and accompany muscle fibers in the tunica muscularis. Our results reveal that EGCs in the esophagus are compatible with this classification system. While GFAP^+^/PLP^+^-glial cells surrounding the myenteric ganglia can be considered to be type I–EGCs (Figure 5A,B), tdT-positive cells directly adjacent to the enteric neurons show similarities to type I-III cells (Figure 4E–H). Meshwork-forming GFAP^−^/PLP^+^–cells, pervading the whole organ, show several branched processes resembling type III glial cells (Figure 4J). In contrast, the intramuscular EGCs that were found in the tunica muscularis and that always lacked GFAP correspond to type IV gliocytes due to their elongated shape and their arrangement between the muscle fibers (Figure 4A–D). These findings can be brought in line with Rao et al., underlining the similarities of EGCs throughout the gastrointestinal tract [30].

Lastly, we found glial cells adjacent to blood vessels in the esophagus. On the one hand, we could detect that not all these cells expressed PLP, as GFAP^+^/PLP^−^ –gliocytes show. On the other hand, these cells varied in their morphology from the ones described above due to their elongated soma as well as their long, gracile processes. As a result, they partially bore a resemblance with type II and type IV glial cells and therefore might possibly represent a fifth type of EGCs in the esophagus.

### 3.4. Enteric Glial Cells as an Immunological Target

In general, there are two possible ways that EGCs can be affected by the immune system: It is conceivable that a direct cell response takes place as the activation of CD8^+^-cells in Crohn’s disease shows [55]. Since EGCs express a battery of glial and myelin markers, it is well possible that these structures can on the other hand serve as immunological targets for a B-cell- and antibody-dependent immune response, respectively. This is even more likely as there are several studies that reveal the presence of autoantibodies against myelin components in MS, including MBP and PLP [56,57,58,59]. Moreover, the clinical relevance of GFAP as a marker for ongoing inflammation in patients with MS is disputed, underlining the involvement of this glial marker in pathological processes [60,61]. To find out how these autoantibodies affect the esophageal ENS and therefore might be involved in the pathogenesis of dysphagia in MS, further studies must be applied. Therefore, the EAE animal model of MS is an eligible possibility for further investigations, as it has already been proven that the ENS represents a potential target for autoimmune processes in MS in the lower gastrointestinal tract [5]. As there are different ways to induce EAE in mice, animals immunized with MP4, a fusion protein of MBP and PLP, resemble the etiopathology of MS more closely in comparison to other EAE models [27,62,63,64]. Hence, we suggest the MP4-EAE-model as an ideal tool for future studies concerning esophageal pathology in MS.

Interestingly, autoimmunity against glial and myelin components is not restricted to demyelinating diseases like MS but is also present in other disease patterns showing gastrointestinal motility disorders and dysphagia. Recent studies demonstrate the presence of MBP-and PLP-autoantibodies in patients with stroke [65,66,67], where more than 50% of all survivors suffer from dysphagia. In addition, GFAP-autoantibodies could not only be found in patients with traumatic brain or spinal cord injury [68,69], but also in those with autoimmune GFAP-astrocytopathy, where at least 20% show autonomic dysfunctions including dysphagia [70]. This fact might open a new perspective on the pathogenesis of dysphagia and shows the possible implication for a variety of neurodegenerative diseases [65,66,67,68,69,70].

In our study, we could identify possible target structures for autoimmune processes in the esophagus. Since the esophageal ENS and its possible implication for dysphagia remains enigmatic, more emphasis should be put on this neglected area of research.

## 4. Materials and Methods

### 4.1. Mice

Immunohistochemical investigations were based on the usage of *n* = 22 C57BL/6J mice of either sex. Furthermore, three tamoxifen-inducible TgN(PLP-CreERT2) mice [71] crossbred with TgH(Rosa26-CAG-lsl-tdTomato) mice [72]) and three double transgenic TgN(hGFAP-EGFP)_GFEC_ mice [73] crossbred with TgN(mPLP-DsRed1)_PRDB_ mice [74]) of either sex were applied. For RT-PCR experiments six additional C57BL/6J mice of either sex were required. All animals were aged between 10 and 15 weeks. All mice were euthanized with a lethal overdose of sodium thiopental (500 mg/kg i.p.). C57BL/6J mice used in the present studies were obtained from The Jackson Laboratories (Charles River, Sulzfeld, Germany) and maintained as inbred lines by full sibling matings under specific pathogen-free conditions at the experimental animal facility (‘Präklinisches Experimentelles Tierzentrum’ (PETZ)) of the University Erlangen-Nürnberg while transgenic mice were held at the animal facility of the Center for Integrative Physiology and Molecular Medicine (CIPMM) of the University of Saarland. PLP-CreERT2 x Rosa26-tdTomato (PLP-CreERT2 x tdT) mice were held in C57BL/6N and GFAP-EGFPxPLP-DsRed1 (GFAP-EGFP x PLP-DsRed1) mice in FVB/N background. Humidity and temperature were maintained at 45–65% and 20–24 °C and the facility kept under a 12-h light-dark cycle. All mice had free access to a standard autoclaved rodent diet (Ssniff Spezialdiäten, Soest, Germany) and autoclaved tap water.

Tamoxifen injection. Tamoxifen solution was prepared as previously described [75]. Briefly, to induce reporter (tdTomato) expression in PLP-CreERT2 mice, tamoxifen (Carbolution, Neunkirchen, Germany) was intraperitoneally injected (10 µg/mL in Mygliol^®^812 (Caesar and Lorentz GmbH, Hilden, Germany), 100 µL/10 g body weight) to mice once per day for three consecutive days. Analysis was executed 14 days after injection.

For the use of all animals the European and German Communities Directive and animal welfare protocols, endorsed by the local government, and the “ARRIVE guidelines for reporting animal research” [76] were followed. Animal experiments and the removal of organs were approved by the local veterinary inspection offices of the University of Erlangen-Nürnberg and the University of Saarland (file reference: TS-99/20-Anatomie I (15.12.1999) and 36/2016 (08.11.2016)).

### 4.2. RT-PCR

#### 4.2.1. Tissue Preparation

Directly after euthanasia, mice were carefully dissected. The cerebrum, cerebellum, brain stem, esophagus, segments of jejunum, and colon ascendens as well as the anterior tibial muscles (both sides) were removed. Tissues were rinsed in sterile Ringer solution (B. Braun, Melsungen, Germany) and snap-frozen in liquid nitrogen. All samples remained stored at −80 °C until RNA extraction.

#### 4.2.2. Processing

For RNA extraction, a modified variant of the single step method according to Chomczynski and Sacchi using TRIzol^®^ reagent (Thermo Fisher Scientific, Carlsbad, CA, USA) was performed. To this end, frozen tissue samples were mechanically pulverized by pestling in a liquid nitrogen-cooled mortar. 50–100 mg of ground tissue were immediately placed in 2 mL RNAse-free tubes and 1 mL of TRIzol^®^ reagent was added. After a 5 min incubation step at room temperature, 0.2 mL of chloroform (Sigma-Aldrich, Taufkirchen, Germany) were added for a 2–3 min incubation at room temperature. Subsequent to a centrifugation step at 12,000× *g* at 4 °C for 15 min, the RNA containing aqueous phase was carefully transferred in a fresh 2 mL RNAse-free tube and 0.5 mL isopropanol (Sigma-Aldrich, Taufkirchen, Germany) were added for another 10 min-incubation at room temperature. A centrifugation step at 12,000× *g* at 4 °C for 10 min was followed by resuspending the RNA precipitate in 1 mL of 75% ethanol (Sigma-Aldrich, Taufkirchen, Germany). After a last centrifugation step at 7500× *g* at 4 °C for 5 min the remaining RNA pellet was air dried and afterwards dissolved in 100 µL of RNAse free DEPC-treated water (Thermo Fisher Scientific, Carlsbad, CA, USA) by heatblock incubation at 57 °C for 15 min. RNA quantification was performed by photometric analysis (BioPhotometer Plus, Eppendorf, Hamburg, Germany). Reverse transcription of all different RNA samples (2 µg per reaction) to cDNA was performed by the usage of High-Capacity cDNA Reverse Transcription Kit (50 U/µL; Thermo Fisher Scientific, Carlsbad, CA, USA) according to manufacturer’s instructions. For gene-specific PCRs 12.5 µL of RedMastermix (2×) Taq PCR-Mastermix (GENAXXON bioscience, Ulm, Germany) were combined with 4 µL of a 1 µM stock solution of each forward and reverse primer, 1 µL template DNA, and 3.5 µL RNAse free DEPC-treated water. PCR reaction was performed in ProFlex PCR System thermal cycler (Thermo Fisher Scientific, Carlsbad, CA, USA). All primers were synthesized at Invitrogen (Thermo Fisher Scientific, Carlsbad, CA, USA). A list of the different primer sequences and the cycle conditions is provided in Table 3.

Agarose gel electrophoresis for all amplified products was carried out on a 2% agarose gel in Tris/acetate/EDTA (TAE)-buffer (pH 8.0) containing GelRed^®^ nucleic acid gel stain (GENAXXON bioscience, Ulm, Germany). Reverse transcriptase PCR products were visualized under ultraviolet light. The housekeeping gene for β-actin was used as loading control.

### 4.3. Immunohistochemistry

For a better overview, the primary and secondary antibodies used are shown in Table 4.

#### 4.3.1. Tissue Preparation and Fixation

Following euthanasia, mice were perfused transcardially with 20 mL of Ringer solution (B. Braun, Melsungen, Germany) prewash, followed by 100 mL 4% phosphate-buffered formaldehyde (pH 7.4).

For frozen sections the cerebellum, the cervical, thoracic, and abdominal portions of the esophagus and both anterior tibial muscles were prepared. For equal full-length division of the esophagus, the thoracic portion, which is approximately twice as long as the other two parts, was divided into an upper and a lower half. Tissues were then postfixed in 4% phosphate-buffered formaldehyde (pH 7.4) for another 5 h at 4 °C and rinsed in phosphate buffer (pH 7.4) at 4 °C overnight. For cryoprotection, tissues were immersed in 12% phosphate-buffered sucrose solution for 24 h at 4 °C. For longtime storage and further processing, tissues were mounted in OCT Embedding Matrix (Carl Roth, Karlsruhe, Germany) and frozen in liquid nitrogen-cooled isopentane.

In those mice, which were intended for whole mount preparations, prior to perfusion, a plastic tubing with a 2 mm outer diameter was inserted in the esophagus in order to distend the organ. After perfusion, the entire esophagus together with the plastic tubing was dissected and postfixed as described above, before rinsing in phosphate buffer (pH 7.4) at 4 °C overnight. In contrast to the other tissues, whole mounts of the esophagus were not frozen but freshly used for immunohistological staining. Therefore, the plastic tubing was removed, the organ was opened longitudinally, and the mucosa, including submucosa, was gently peeled off.

#### 4.3.2. Frozen Sections

For all frozen section stainings, 12-µm-thick cryostat sections were cut using a Leica CM1900 cryostat (Leica, Wetzlar, Germany). Slices were then mounted on poly-L-lysine coated slides and air-dried for at least one hour at RT. A hydrophobic barrier around the specimens on the slides was provided by drawn lines with ImmEdge™ Hydrophobic Barrier Pen (Vector Laboratories, Burlingame, CA, USA). The preincubation solution contained a mixture of 1% bovine serum albumin (BSA; Carl Roth, Karlsruhe, Germany), 0.5% Triton^®^ × 100 (Carl Roth, Karlsruhe, Germany), Tris-buffered saline (TBS; 0.05 M, pH 7.3), and, depending on the host species of the used secondary antibody, 5% normal donkey serum (Jackson ImmunoResearch, West Grove, PA, USA) and/or 5% normal goat serum (Jackson ImmunoResearch, West Grove, PA, USA). Before and after preincubation, which was performed for one hour at room temperature, slides were washed in TBS. In protocols where the PLP-antibody was applied, an extra preincubation with a mixture of TBS and 10% Triton^®^ × 100 (Carl Roth, Karlsruhe, Germany) was prepended for 15 min to the common preincubation. Primary antibody incubation was performed over night at room temperature, followed by another rinsing step in TBS for 15 min. Secondary antibody incubation was subsequently performed for one hour at room temperature, completed with a 15 min washing step in TBS. For muscular tissues, i.e., esophagus- and anterior tibial muscle-sections, a 20 min incubation with fluorochrome-tagged α-BT was added, in order to label motor endplates. If necessary, a nuclear staining with Hoechst was appended in the end for 10 min. After a final wash in TBS for 15 min, all sections were cover slipped with a 1:1 mixture of TBS-glycerol (pH 8.6). The exact setting of primary and secondary antibodies for each staining is shown in Table 1.

#### 4.3.3. Whole Mounts

After the removal of the mucosal tissue, whole-mount preparations were preincubated at room temperature for two hours on a shaker with a preincubation solution consisting out of 1% BSA (Carl Roth, Karlsruhe, Germany), 2.5% Triton^®^ × 100 (Carl Roth, Karlsruhe, Germany), TBS (0.05 M, pH 7.3), 0.05% Thimerosal (Carl Roth, Karlsruhe, Germany), and, depending on the host species of the used secondary antibody, 5% normal donkey serum (Jackson ImmunoResearch, West Grove, PA, USA) and/or 5% normal goat serum (Jackson ImmunoResearch, West Grove, PA, USA). In protocols where the PLP-antibody was applied, an extra preincubation with a mixture of TBS and 10% Triton^®^ × 100 (Carl Roth, Karlsruhe, Germany) was prepended for 15 min to the common preincubation. Whole-mount tissues were then rinsed in TBS for 10 min and subsequently put into the specific primary antibody incubation for three days at 4 °C on a shaker. Afterwards, a washing step in TBS for one day at 4 °C on a shaker was performed, before the secondary antibody incubation was started for four hours at room temperature on a shaker, followed by an additional rinsing step in TBS for one day at 4 °C on a shaker. In the case of motor endplate-labelling with fluorochrome-tagged α-BT, incubation time was prolonged to one hour at room temperature. For nuclear staining, an incubation with Hoechst was appended in the end for 10 min. After a final wash in TBS for 15 min, all sections were cover slipped with a 1:1 mixture of TBS-glycerol (pH 8.6). The exact setting of primary and secondary antibodies for each staining is shown in Table 2.

#### 4.3.4. Control Experiments

The specificity of the immunohistochemical reactions was assessed by replacing the primary antibody with TBS or the respective host serum as negative controls or by pre-absorbing the antibody against Calbindin D28k, ChAT, PGP 9.5 and Synaptophysin with its respective antigen (Calbindin D28k: Synaptic Systems, Göttingen, Germany; ChAT: Millipore, Billerica, MA, USA; PGP 9.5: Fitzgerald Ind., Acton, MA, USA; Synaptophysin: Synaptic Systems, Göttingen, Germany). For specificity control of GFAP, PLP, and MBP, we used cryosections of the cerebellum as positive controls as described above. Specificity of DsRed and GFP antibodies was confirmed by combined cell-type specific expression of PLP (red fluorescence in transgenic PLP-CreERT2 x tdT mice) and GFAP (green fluorescence in transgenic GFAP-EGFP x PLP-DsRed1 mice).

### 4.4. Image Aquisition

All stainings were evaluated using a fluorescence microscope with a confocal laser scanning system (Nikon Eclipse E1000-M; Nikon Digital Eclipse C1 with software EZ-C1 3.91; Tokyo, Japan) equipped with the digital camera system Nikon Digital Sight DS-2MBWc. The system provided a quadruple laser configuration, consisting of a 405 nm Diode-Laser (Coherent: CUBE 405-100C), a 488 nm and a 543 nm Solid-State-Laser (both from Coherent, Santa Clara, CA, USA) and a 642 nm Diode-Laser (Melles-Griot, Carlsbad, CA, USA). In order to reduce unspecific background fluorescence, a BIO1-Filterset (DAPI/Cy5 for C1-Detector; AHF Analysentechnik, Tübingen, Germany) was additionally installed.

Dry objective lenses with 20× and 40× magnification and a numerical aperture of 0.75 and 0.95, respectively, were used in combination with an electronical zoom factor from 1.0 to 4.0. To obtain all-in-focus images, up to 18 optical sections were taken at intervals of 0.5–1 µm in the z-axis and electronically superimposed. Image processing was performed with Nikon Free Viewer software (EZ-C1 3.91) and Volocity Demo 6.1.1 (PerkinElmer, Waltham, MA, USA), brightness and contrast were adjusted by the usage of Adobe Photoshop CS6 (Adobe Systems, San Josè, CA, USA) and layout was configured with CorelDRAW X7 (Ottawa, ON, Canada).

## 5. Conclusions

The present study provides detailed data of myelin and glial structures in the murine esophagus, which are possible targets for autoimmune processes in this organ. Besides PLP-, GFAP- and MBP-positive Schwann cells surrounding vagal nerve fibers and PLP- and GFAP-positive glial cells surrounding myenteric neurons, we observed numerous PLP-positive glial cells forming a network in the esophageal wall. It is tempting to speculate, that subtypes of these enteric glial cells, which are closely associated with striated muscle fibers, are involved in the enteric co-innervation of esophageal motor endplates at the peripheral level. It will be worthwhile, to study alterations of these PLP-positive enteric glial cells in mouse models of MS and other demyelinating diseases and to delineate their possible participation in the pathogenesis of dysphagia.

## Figures and Tables

**Table 1:** Synopsis of applied staining protocols for frozen sections including each antibody setup.

N°	Staining Protocol	Antibody Setup
**①**	Quadruple staining of PLP, Calbindin D28k, MBP and Hoechst for Cerebellar Tissue	**Primary Antibodies**
		Rat anti-PLP Guinea pig anti-Calbindin D28k Rabbit anti-MBP
		**Secondary Antibodies**
		Donkey anti-rat Alexa 488 Goat anti-guinea pig Alexa 555 Donkey anti-rabbit Alexa 647 Hoechst
**②**	Quadruple staining of PLP, Calbindin D28k, GFAP and Hoechst for Cerebellar Tissue	**Primary Antibodies**
		Rat anti-PLP Guinea pig anti-Calbindin D28k Rabbit anti-GFAP
		**Secondary Antibodies**
		Donkey anti-rat Alexa 488 Goat anti-guinea pig Alexa 555 Donkey anti-rabbit Alexa 647 Hoechst
**③**	Triple Staining of GFAP, Synaptophysin and α-BT for Skeletal Muscle and Esophagus	**Primary Antibodies**
		Rabbit anti-GFAP Guinea pig anti-Synaptophysin
		**Secondary Antibodies and Toxins**
		Donkey anti-rabbit Alexa 488 Donkey anti-guinea pig Alexa 647 α-Bungarotoxin Alexa 555
**④**	Triple staining of MBP, Synaptophysin and α-BT for Skeletal Muscle	**Primary Antibodies**
		Rabbit anti-MBP Guinea pig anti-Synaptophysin
		**Secondary Antibodies and Toxins**
		Donkey anti-rabbit Alexa 488 Donkey anti-guinea pig Alexa 647 α-Bungarotoxin Alexa 555
**⑤**	Quadruple staining of PLP, Synaptophysin, α-BT and Hoechst for Skeletal Muscle and Esophagus	**Primary Antibodies**
		Rat anti-PLP Guinea pig anti-Synaptophysin
		**Secondary Antibodies and Toxins**
		Donkey anti-rat Alexa 488 Donkey anti-guinea pig Alexa 647 α-Bungarotoxin Alexa 555 Hoechst
**⑥**	Triple Staining of ChAT, MBP and α-BT for Skeletal Muscle	**Primary Antibodies**
		Goat anti-ChAT Rabbit anti-MBP
		**Secondary Antibodies and Toxins**
		Donkey anti-goat Alexa 647 Donkey anti-rabbit Alexa 488 α-Bungarotoxin Alexa 555
**⑦**	Triple Staining of ChAT, Synaptophysin and α-BT for Skeletal Muscle	**Primary Antibodies**
		Goat anti-ChAT Guinea pig anti-Synaptophysin
		**Secondary Antibodies and Toxins**
		Donkey anti-goat Alexa 488 Donkey anti-guinea pig Alexa 647 α-Bungarotoxin Alexa 555
**⑧**	Triple Staining of βIII-tubulin, MBP and α-BT for Skeletal Muscle	**Primary Antibodies**
		Rabbit anti-βIII-tubulin Chicken anti-MBP
		**Secondary Antibodies and Toxins**
		Donkey anti-rabbit Alexa 488 Goat anti-chicken Alexa 647 α-Bungarotoxin Alexa 555

**Table 2:** Synopsis of applied staining protocols for whole mounts including each antibody setup.

N°	Staining Protocol	Antibody Setup
**①**	Quadruple Staining of MBP, ChAT, α-BT and Hoechst	**Primary Antibodies**
		Rabbit anti-MBP Goat anti-ChAT
		**Secondary Antibodies and Toxins**
		Donkey anti-rabbit Alexa 488 Donkey anti-Goat Alexa 647 α-Bungarotoxin Alexa 555 Hoechst
**②**	Triple Staining of DsRed, PLP and Protein Gene Product 9.5 (PGP 9.5) in PLP-CreERT2 x tdT mice	**Primary Antibodies**
		Rabbit anti-DsRed (This antibody also binds to tdTomato) Rat anti-PLP Guinea pig anti-PGP 9.5
		**Secondary Antibodies**
		Donkey anti-rabbit Alexa 647 Donkey anti-rat Alexa 488 Donkey anti-guinea pig DYE 405
**③**	Triple Staining of GFAP, PGP 9.5 and Hoechst	**Primary Antibodies**
		Rabbit anti-GFAP Guinea pig anti-PGP 9.5
		**Secondary Antibodies and Toxins**
		Donkey anti-rabbit Alexa 488 Goat anti-guinea pig Alexa 555 Hoechst
**④**	Double Staining of GFP and DsRed in GFAP-EGFP x PLP-DsRed1 Mice	**Primary Antibodies**
		Chicken anti-GFP Rabbit anti-DsRed
		**Secondary Antibodies and Toxins**
		Goat anti-chicken Alexa 647 Donkey anti-rabbit DYE 405
**⑤**	Triple staining of βIII-Tubulin, MBP and α-BT	**Primary Antibodies**
		Rabbit anti- βIII-tubulin Chicken anti-MBP
		**Secondary Antibodies and Toxins**
		Donkey anti-rabbit Alexa 488 Donkey anti-chicken Alexa 647 α-Bungarotoxin Alexa 555

**Table 3:** Overview of used primer pairs for RT-PCR including cycle conditions.

Gene	Forward Primer Reverse Primer Primer Reference	Cycle Conditions			
***β*** ***-Actin*** **(154 bp)**	(F) 5′-GGCTGTATTCCCCTCCATCG-3′ (R) 5′-CCAGTTGGTAACAATGCCATGT-3′ Self-Designed	**Initial Denaturation**		10 min	95 °C
		35 Cycles	Denaturation	45 s	95 °C
			Annealing	30 s	57 °C
			Extension	45 s	72 °C
		Final extension		10 min	72 °C
***GFAP*** **(199 bp)**	(F) 5′-CAACGTTAAGCTAGCCCTGGACAT-3′ (R) 5′-CTCACCATCCCGCATCTCCACAGT-3′ Shi et al. [77]	Initial denaturation		10 min	95 °C
		35 Cycles	Denaturation	45 s	95 °C
			Annealing	30 s	60 °C
			Extension	45 s	72 °C
		Final extension		10 min	72 °C
***PLP*** **(218 bp)**	(F) 5′-AGCGGGTGTGTCATTGTTTGGGAA-3′ (R) 5′-ACCATACATTCTGGCATCAGCGCA-3′ Chew et al. [78]	Initial denaturation		10 min	95 °C
		35 Cycles	Denaturation	45 s	95 °C
			Annealing	30 s	58 °C
			Extension	45 s	72 °C
		Final extension		10 min	72 °C
***MBP*** **(342–642 bp)**	(F) 5′-ATGGCATCACAGAAGAGACC-3′ (R) 5′-CATGGGAGATCCAGAGCGGC-3′ Ye et al. [79]	Initial denaturation		10 min	95 °C
		35 Cycles	Denaturation	45 s	95 °C
			Annealing	30 s	56 °C
			Extension	45 s	72 °C
		Final extension		10 min	72 °C
***MAG*** **(355–400 bp)**	(F) 5′-CTCTATGGCACCCAGAGCCT-3′ (R) 5′-TGTCCTTGGTGGGTCGTTTT-3′ Ye et al. [79]	Initial denaturation		10 min	95 °C
		35 Cycles	Denaturation	45 s	95 °C
			Annealing	30 s	56 °C
			Extension	45 s	72 °C
		Final extension		10 min	72 °C
***OSP*** **(339 bp)**	(F) 5′-GATTGGCATCATCGTCACAACG-3′ (R) 5′-AGCCAGCAGAATAAGGAGCACC-3′ Hellani et al. [80]	Initial denaturation		10 min	95 °C
		35 Cycles	Denaturation	45 s	95 °C
			Annealing	30 s	50 °C
			Extension	45 s	72 °C
		Final extension		10 min	72 °C
***MOG*** **(841 bp)**	(F) 5′-GACCTCAGCTTGGCCTGACCC-3′ (R) 5′-TGCTGGGCTCTCCTTCCGC-3′ Delarasse et al. [81]	Initial denaturation		5 min	94 °C
		35 Cycles	Denaturation	1 min	95 °C
			Annealing	1 min	66 °C
			Extension	3 min	72 °C
		Final extension		5 min	72 °C

**Table 4:** Characterization of antibodies and toxins used for immunohistochemical staining.

Primary Antibodies	Host Species	Dilution	Source (Catalogue Number)
**Calbindin D28k**	Guinea pig	1:100	Synaptic Systems Göttingen, Germany (214 004)
**ChAT**	Goat	1:40	Millipore Temecula, CA, USA (AB144P–1ML)
**DsRed**	Rabbit	1:1000	Takara Bio Mountain View, CA, USA (632496)
**GFAP**	Rabbit	1:800–1:2000	Dako Glostrup, Denmark (Z0334)
**GFP**	Chicken	1:1000	ThermoFisher Waltham, MA, USA (A10262)
**MBP**	Rabbit	1:200–1:500	Abcam Cambridge, UK (ab40390)
**MBP**	Chicken	1:500–1:3000	Abcam Cambridge, UK (ab134018)
**PGP 9.5**	Guinea pig	1:500	Fitzgerald Ind. Acton, MA, USA (20R–PG011)
**PLP (PLP1)**	Rat	1:1000	Kind gift from Wendy B. Macklin
**Synaptophysin**	Guinea pig	1:1000	Synaptic Systems Göttingen, Germany (101 004)
**β** **III-Tubulin**	Rabbit	1:500–1:4000	Abcam Cambridge, UK (ab18207)
**Secondary Antibodies and Toxins**		**Dilution**	**Source (Catalogue Number)**
**Donkey Anti-Chicken Alexa 647**		1:1000	Jackson ImmunoResearch West Grove, PA, USA (703–605–155)
**Goat Anti-Chicken Alexa 647**		1:1000	Jackson ImmunoResearch West Grove, PA, USA (103–605–155)
**Donkey Anti-Goat Alexa 488**		1:1000	Molecular Probes Eugene, OR, USA (A11055)
**Donkey Anti-Goat Alexa 647**		1:1000	Molecular Probes Eugene, OR, USA (A21447)
**Donkey Anti-Guinea Pig DYE 405**		1:200	Jackson ImmunoResearch West Grove, PA, USA (706–475–148)
**Goat Anti-Guinea Pig Alexa 555**		1:1000	Molecular Probes Eugene, OR, USA (A–21435)
**Donkey Anti-Guinea Pig Alexa 647**		1:1000	Jackson ImmunoResearch West Grove, PA, USA (706–605–148)
**Donkey Anti-Rabbit DYE 405**		1:200	Jackson ImmunoResearch West Grove, PA, USA (711–475–152)
**Donkey Anti-Rabbit Alexa 488**		1:1000	ThermoFisher Waltham, MA, USA (A–21206)
**Donkey Anti-Rabbit Alexa 647**		1:1000	Molecular Probes Eugene, OR, USA (A31573)
**Donkey Anti-Rat Alexa 488**		1:1000	ThermoFisher Waltham, MA, USA (A–21208)
**α** **-Bungarotoxin Alexa 555**		1:1000	Molecular Probes Eugene, OR, USA (B35451)
**Hoechst**		1:1000	Sigma-Aldrich St. Louis, MO, USA (H6024)

## Data Availability

The data presented in this study are available on request from the corresponding author.

## Supplementary Materials

Supplementary materials can be found at https://www.mdpi.com/1422-0067/22/6/3233/s1. Supplementary Figure S1: Control stainings for PLP, MBP and GFAP in the murine cerebellum, Supplementary Figure S2: Expression of GFAP, MBP and PLP in the NMJ of the tibialis anterior muscle, Supplementary Figure S3: Expression of βIII-tubulin and MBP in nerve fiber bundles, around blood vesels and in the NMJ of the tibialis anterior muscle, Supplementary Figure S4: Positive control stainings of ChAT and comparison of ChAT and synaptophysin distribution in the tibialis anterior muscle.

## Abbreviations

BSA	Bovine serum albumin
CALB	Calbindin (D28k)
cDNA	Complementary DNA
ChAT	Choline Acetyltransferase
CNS	Central nervous system
DEPC	Diethyl pyrocarbonate
DsRed	Discosoma sp. red fluorescent protein
EAE	Experimental autoimmune encephalomyelitis
EDTA	Ethylenediaminetetraacetic acid
EGC	Enteric glial cell
EGFP	Enhanced green fluorescent protein
ENS	Enteric nervous system
GFAP	Glial fibrillary acidic protein
GFP	Green fluorescent protein
MAG	Myelin-associated glycoprotein (also: Siglec-4)
MBP	Myelin basic protein
MOG	Myelin oligodendrocyte glycoprotein
MS	Multiple sclerosis
NMJ	Neuromuscular junction
OSP	Oligodendrocyte-specific protein (also: Claudin-11)
PETZ	Präklinisches Experimentelles Tierzentrum; animal facility of the University Erlangen-Nürnberg
PGP 9.5	Protein gene product 9.5
PLP / PLP1	Proteolipid protein 1 (also: lipohilin)
PNS	Peripheral nervous system
PSC	Perisynaptic Schwann cell
RT-PCR	Reverse transcriptase polymerase chain reaction
TAE	Tris/acetate/EDTA-buffer
TBS	Tris-buffered saline
tdT	tdTomato
α-BT	α-Bungarotoxin

## References

[1] Compston A., Coles A. (2008). Multiple sclerosis. Lancet.

[2] Mahad D.H., Trapp B.D., Lassmann H. (2015). Pathological mechanisms in progressive multiple sclerosis. Lancet Neurol..

[3] Katz Sand I. (2015). Classification, diagnosis, and differential diagnosis of multiple sclerosis. Curr. Opin. Neurol..

[4] Weber M.S., Hemmer B., Cepok S. (2011). The role of antibodies in multiple sclerosis. Biochim. Biophys. Acta.

[5] Wunsch M., Jabari S., Voussen B., Enders M., Srinivasan S., Cossais F., Wedel T., Boettner M., Schwarz A., Weyer L. (2017). The enteric nervous system is a potential autoimmune target in multiple sclerosis. Acta Neuropathol..

[6] Spear E.T., Holt E.A., Joyce E.J., Haag M.M., Mawe S.M., Hennig G.W., Lavoie B., Applebee A.M., Teuscher C., Mawe G.M. (2018). Altered gastrointestinal motility involving autoantibodies in the experimental autoimmune encephalomyelitis model of multiple sclerosis. Neurogastroenterol. Motil..

[7] Levinthal D.J., Rahman A., Nusrat S., O’Leary M., Heyman R., Bielefeldt K. (2013). Adding to the burden: Gastrointestinal symptoms and syndromes in multiple sclerosis. Mult. Scler. Int..

[8] Levinthal D.J., Bielefeldt K. (2017). Systematic review and meta-analysis: Gastric electrical stimulation for gastroparesis. Auton. Neurosci..

[9] Preziosi G., Raptis D.A., Raeburn A., Thiruppathy K., Panicker J., Emmanuel A. (2013). Gut dysfunction in patients with multiple sclerosis and the role of spinal cord involvement in the disease. Eur. J. Gastroenterol. Hepatol..

[10] Calcagno P., Ruoppolo G., Grasso M.G., de Vincentiis M., Paolucci S. (2002). Dysphagia in multiple sclerosis - prevalence and prognostic factors. Acta Neurol. Scand..

[11] Tassorelli C., Bergamaschi R., Buscone S., Bartolo M., Furnari A., Crivelli P., Alfonsi E., Alberici E., Bertino G., Sandrini G. (2008). Dysphagia in multiple sclerosis: From pathogenesis to diagnosis. Neurol. Sci..

[12] De Pauw A., Dejaeger E., D’hooghe B., Carton H. (2002). Dysphagia in multiple sclerosis. Clin. Neurol. Neurosurg..

[13] Alali D., Ballard K., Vucic S., Bogaardt H. (2018). Dysphagia in Multiple Sclerosis: Evaluation and Validation of the DYMUS Questionnaire. Dysphagia.

[14] Barkhof F., Koeller K.K., Hodler J., Kubik-Huch R.A., von Schulthess G.K. (2020). Demyelinating Diseases of the CNS (Brain and Spine). Diseases of the Brain, Head and Neck, Spine 2020-2023: Diagnostic Imaging.

[15] Love S. (2006). Demyelinating diseases. J. Clin. Pathol..

[16] Alali D., Ballard K., Bogaardt H. (2016). Treatment Effects for Dysphagia in Adults with Multiple Sclerosis: A Systematic Review. Dysphagia.

[17] Kim J.-S., Sung H.-Y. (2015). Gastrointestinal Autonomic Dysfunction in Patients with Parkinson’s Disease. J. Mov. Disord..

[18] Verne G.N., Sallustio J.E., Eaker E.Y. (1997). Anti-myenteric neuronal antibodies in patients with achalasia. A prospective study. Dig. Dis. Sci..

[19] Neuhuber W.L., Wörl J., Berthoud H.R., Conte B. (1994). NADPH-diaphorase-positive nerve fibers associated with motor endplates in the rat esophagus: New evidence for co-innervation of striated muscle by enteric neurons. Cell Tissue Res..

[20] Wörl J., Mayer B., Neuhuber W.L. (1994). Nitrergic innervation of the rat esophagus: Focus on motor endplates. J. Auton. Nerv. Syst..

[21] Wörl J., Neuhuber W.L. (2005). Enteric co-innervation of motor endplates in the esophagus: State of the art ten years after. Histochem. Cell Biol..

[22] Neuhuber W.L., Wörl J. (2016). Enteric co-innervation of striated muscle in the esophagus: Still enigmatic?. Histochem. Cell Biol..

[23] Kuramoto H., Yoshimura R., Sakamoto H., Kadowaki M. (2019). Regional variations in the number distribution of intrinsic myenteric neurons and coinnervated motor endplates on the striated muscles in the rat esophagus. Auton. Neurosci..

[24] Furness J.B. (2012). The enteric nervous system and neurogastroenterology. Nat. Rev. Gastroenterol. Hepatol..

[25] Chadi G., Gomide V.C., Rodrigues de Souza R., Scabello R.T., Maurício da Silva C. (2004). Basic fibroblast growth factor, neurofilament, and glial fibrillary acidic protein immunoreactivities in the myenteric plexus of the rat esophagus and colon. J. Morphol..

[26] Raab M., Neuhuber W.L. (2004). Intraganglionic laminar endings and their relationships with neuronal and glial structures of myenteric ganglia in the esophagus of rat and mouse. Histochem. Cell Biol..

[27] Kuerten S., Lichtenegger F.S., Faas S., Angelov D.N., Tary-Lehmann M., Lehmann P.V. (2006). MBP-PLP fusion protein-induced EAE in C57BL/6 mice. J. Neuroimmunol..

[28] Robinson A.P., Harp C.T., Noronha A., Miller S.D. (2014). The experimental autoimmune encephalomyelitis (EAE) model of MS: Utility for understanding disease pathophysiology and treatment. Handb. Clin. Neurol..

[29] Mørkholt A.S., Kastaniegaard K., Trabjerg M.S., Gopalasingam G., Niganze W., Larsen A., Stensballe A., Nielsen S., Nieland J.D. (2018). Identification of brain antigens recognized by autoantibodies in experimental autoimmune encephalomyelitis-induced animals treated with etomoxir or interferon-β. Sci. Rep..

[30] Rao M., Nelms B.D., Dong L., Salinas-Rios V., Rutlin M., Gershon M.D., Corfas G. (2015). Enteric glia express proteolipid protein 1 and are a transcriptionally unique population of glia in the mammalian nervous system. Glia.

[31] Grundmann D., Loris E., Maas-Omlor S., Huang W., Scheller A., Kirchhoff F., Schäfer K.-H. (2019). Enteric Glia: S100, GFAP, and Beyond. Anat. Rec..

[32] Mayer M.C., Meinl E. (2012). Glycoproteins as targets of autoantibodies in CNS inflammation: MOG and more. Ther. Adv. Neurol. Disord..

[33] Quarles R.H. (2007). Myelin-associated glycoprotein (MAG): Past, present and beyond. J. Neurochem..

[34] Pascual-Goñi E., Martín-Aguilar L., Lleixà C., Martínez-Martínez L., Simón-Talero M.J., Díaz-Manera J., Cortés-Vicente E., Rojas-García R., Moga E., Juárez C. (2019). Clinical and laboratory features of anti-MAG neuropathy without monoclonal gammopathy. Sci. Rep..

[35] Bronstein J.M., Micevych P.E., Chen K. (1997). Oligodendrocyte-specific protein (OSP) is a major component of CNS myelin. J. Neurosci. Res..

[36] Yu Y., Fu Y., Watson C. (2014). The inferior olive of the C57BL/6J mouse: A chemoarchitectonic study. Anat. Rec..

[37] Grosche J., Matyash V., Möller T., Verkhratsky A., Reichenbach A., Kettenmann H. (1999). Microdomains for neuron-glia interaction: Parallel fiber signaling to Bergmann glial cells. Nat. Neurosci..

[38] Triolo D., Dina G., Lorenzetti I., Malaguti M., Morana P., Del Carro U., Comi G., Messing A., Quattrini A., Previtali S.C. (2006). Loss of glial fibrillary acidic protein (GFAP) impairs Schwann cell proliferation and delays nerve regeneration after damage. J. Cell Sci..

[39] Ko C.-P., Sugiura Y., Feng Z., Armati P. (2007). The biology of perisynaptic (terminal) Schwann cells. The Biology of Schwann Cells.

[40] Barik A., Li L., Sathyamurthy A., Xiong W.-C., Mei L. (2016). Schwann Cells in Neuromuscular Junction Formation and Maintenance. J. Neurosci..

[41] Armati P.J., Mathey E.K. (2013). An update on Schwann cell biology--immunomodulation, neural regulation and other surprises. J. Neurol. Sci..

[42] Sugiura Y., Lin W. (2011). Neuron-glia interactions: The roles of Schwann cells in neuromuscular synapse formation and function. Biosci. Rep..

[43] Feng Z., Ko C.-P. (2008). Schwann cells promote synaptogenesis at the neuromuscular junction via transforming growth factor-beta1. J. Neurosci..

[44] Auld D.S., Robitaille R. (2003). Perisynaptic Schwann cells at the neuromuscular junction: Nerve- and activity-dependent contributions to synaptic efficacy, plasticity, and reinnervation. Neuroscientist.

[45] Gruber H. (1968). Uber Struktur und Innervation der quergestreiften Muskulatur des Oesophagus der Ratte. Z. Zellforsch. Mikrosk. Anat..

[46] Samarasinghe D.D. (1972). Some observations on the innervation of the striated muscle in the mouse oesophagus–an electron microscopy study. J. Anat..

[47] Berthoud H.R., Patterson L.M., Neumann F., Neuhuber W.L. (1997). Distribution and structure of vagal afferent intraganglionic laminar endings (IGLEs) in the rat gastrointestinal tract. Anat. Embryol..

[48] Powley T.L., Baronowsky E.A., Gilbert J.M., Hudson C.N., Martin F.N., Mason J.K., McAdams J.L., Phillips R.J. (2013). Vagal afferent innervation of the lower esophageal sphincter. Auton. Neurosci..

[49] Neuhuber W.L. (1987). Sensory vagal innervation of the rat esophagus and cardia: A light and electron microscopic anterograde tracing study. J. Auton. Nerv. Syst..

[50] Sengupta J.N. (2006). Esophageal sensory physiology. GI Motil. Online.

[51] Clouse R.E., Richter J.E., Heading R.C., Janssens J., Wilson J.A. (1999). Functional esophageal disorders. Gut.

[52] Richter J.E., Barish C.F., Castell D.O. (1986). Abnormal sensory perception in patients with esophageal chest pain. Gastroenterology.

[53] Hanani M., Reichenbach A. (1994). Morphology of horseradish peroxidase (HRP)-injected glial cells in the myenteric plexus of the guinea-pig. Cell Tissue Res..

[54] Gulbransen B.D., Sharkey K.A. (2012). Novel functional roles for enteric glia in the gastrointestinal tract. Nat. Rev. Gastroenterol. Hepatol..

[55] Cornet A., Savidge T.C., Cabarrocas J., Deng W.L., Colombel J.F., Lassmann H., Desreumaux P., Liblau R.S. (2001). Enterocolitis induced by autoimmune targeting of enteric glial cells: A possible mechanism in Crohn’s disease?. Proc. Natl. Acad. Sci. USA.

[56] Olsson T., Baig S., Höjeberg B., Link H. (1990). Antimyelin basic protein and antimyelin antibody-producing cells in multiple sclerosis. Ann. Neurol..

[57] Link H., Baig S., Olsson O., Jiang Y.-P., Höjeberg B., Olsson T. (1990). Persistent anti-myelin basic protein IgG antibody response in multiple sclerosis cerebrospinal fluid. J. Neuroimmunol..

[58] Sellebjerg F.T., Frederiksen J.L., Olsson T. (1994). Anti-myelin basic protein and anti-proteolipid protein antibody-secreting cells in the cerebrospinal fluid of patients with acute optic neuritis. Arch. Neurol..

[59] Sun J.B., Olsson T., Wang W.Z., Xiao B.G., Kostulas V., Fredrikson S., Ekre H.P., Link H. (1991). Autoreactive T and B cells responding to myelin proteolipid protein in multiple sclerosis and controls. Eur. J. Immunol..

[60] Abdelhak A., Huss A., Kassubek J., Tumani H., Otto M. (2018). Serum GFAP as a biomarker for disease severity in multiple sclerosis. Sci. Rep..

[61] Kassubek R., Gorges M., Schocke M., Hagenston V.A.M., Huss A., Ludolph A.C., Kassubek J., Tumani H. (2017). GFAP in early multiple sclerosis: A biomarker for inflammation. Neurosci. Lett..

[62] Kuerten S., Javeri S., Tary-Lehmann M., Lehmann P.V., Angelov D.N. (2008). Fundamental differences in the dynamics of CNS lesion development and composition in MP4- and MOG peptide 35-55-induced experimental autoimmune encephalomyelitis. Clin. Immunol..

[63] Kuerten S., Kostova-Bales D.A., Frenzel L.P., Tigno J.T., Tary-Lehmann M., Angelov D.N., Lehmann P.V. (2007). MP4- and MOG:35-55-induced EAE in C57BL/6 mice differentially targets brain, spinal cord and cerebellum. J. Neuroimmunol..

[64] Recks M.S., Stormanns E.R., Bader J., Arnhold S., Addicks K., Kuerten S. (2013). Early axonal damage and progressive myelin pathology define the kinetics of CNS histopathology in a mouse model of multiple sclerosis. Clin. Immunol..

[65] Becker K.J., Tanzi P., Zierath D., Buckwalter M.S. (2016). Antibodies to myelin basic protein are associated with cognitive decline after stroke. J. Neuroimmunol..

[66] Gee J.M., Kalil A., Thullbery M., Becker K.J. (2008). Induction of immunologic tolerance to myelin basic protein prevents central nervous system autoimmunity and improves outcome after stroke. Stroke.

[67] Shibata D., Cain K., Tanzi P., Zierath D., Becker K. (2012). Myelin basic protein autoantibodies, white matter disease and stroke outcome. J. Neuroimmunol..

[68] Zhang Z., Zoltewicz J.S., Mondello S., Newsom K.J., Yang Z., Yang B., Kobeissy F., Guingab J., Glushakova O., Robicsek S. (2014). Human traumatic brain injury induces autoantibody response against glial fibrillary acidic protein and its breakdown products. PLoS ONE.

[69] Hergenroeder G.W., Moore A.N., Schmitt K.M., Redell J.B., Dash P.K. (2016). Identification of autoantibodies to glial fibrillary acidic protein in spinal cord injury patients. Neuroreport.

[70] Kunchok A., Zekeridou A., McKeon A. (2019). Autoimmune glial fibrillary acidic protein astrocytopathy. Curr. Opin. Neurol..

[71] Leone D.P., Genoud S., Atanasoski S., Grausenburger R., Berger P., Metzger D., Macklin W.B., Chambon P., Suter U. (2003). Tamoxifen-inducible glia-specific Cre mice for somatic mutagenesis in oligodendrocytes and Schwann cells. Mol. Cell Neurosci..

[72] Madisen L., Zwingman T.A., Sunkin S.M., Oh S.W., Zariwala H.A., Gu H., Ng L.L., Palmiter R.D., Hawrylycz M.J., Jones A.R. (2010). A robust and high-throughput Cre reporting and characterization system for the whole mouse brain. Nat. Neurosci..

[73] Lalo U., Pankratov Y., Kirchhoff F., North R.A., Verkhratsky A. (2006). NMDA receptors mediate neuron-to-glia signaling in mouse cortical astrocytes. J. Neurosci..

[74] Hirrlinger P.G., Scheller A., Braun C., Quintela-Schneider M., Fuss B., Hirrlinger J., Kirchhoff F. (2005). Expression of reef coral fluorescent proteins in the central nervous system of transgenic mice. Mol. Cell. Neurosci..

[75] Jahn H.M., Kasakow C.V., Helfer A., Michely J., Verkhratsky A., Maurer H.H., Scheller A., Kirchhoff F. (2018). Refined protocols of tamoxifen injection for inducible DNA recombination in mouse astroglia. Sci. Rep..

[76] Kilkenny C., Browne W.J., Cuthill I.C., Emerson M., Altman D.G. (2010). Improving bioscience research reporting: The ARRIVE guidelines for reporting animal research. PLoS Biol..

[77] Shi X., Yan C., Liu B., Yang C., Nie X., Wang X., Zheng J., Wang Y., Zhu Y. (2015). miR-381 Regulates Neural Stem Cell Proliferation and Differentiation via Regulating Hes1 Expression. PLoS ONE.

[78] Chew L.-J., Coley W., Cheng Y., Gallo V. (2010). Mechanisms of regulation of oligodendrocyte development by p38 mitogen-activated protein kinase. J. Neurosci..

[79] Ye P., Bagnell R., D’Ercole A.J. (2003). Mouse NG2 + Oligodendrocyte Precursors Express mRNA for Proteolipid Protein But Not Its DM-20 Variant: A Study of Laser Microdissection-Captured NG2 + Cells. J. Neurosci..

[80] Hellani A., Ji J., Mauduit C., Deschildre C., Tabone E., Benahmed M. (2000). Developmental and hormonal regulation of the expression of oligodendrocyte-specific protein/claudin 11 in mouse testis. Endocrinology.

[81] Delarasse C., Daubas P., Mars L.T., Vizler C., Litzenburger T., Iglesias A., Bauer J., Della Gaspera B., Schubart A., Decker L. (2003). Myelin/oligodendrocyte glycoprotein–deficient (MOG-deficient) mice reveal lack of immune tolerance to MOG in wild-type mice. J. Clin. Investig..

