# Supplementary material for: Murine Esophagus Expresses Glial-Derived Central Nervous System Antigens
Source: Int J Mol Sci. 2021 Mar 22;22(6):3233. doi: 10.3390/ijms22063233 (PMC8004938; doi:10.3390/ijms22063233)
Supplement: Supplementary file 1 [file ijms-22-03233-s001.pdf]

Supplementary Materials

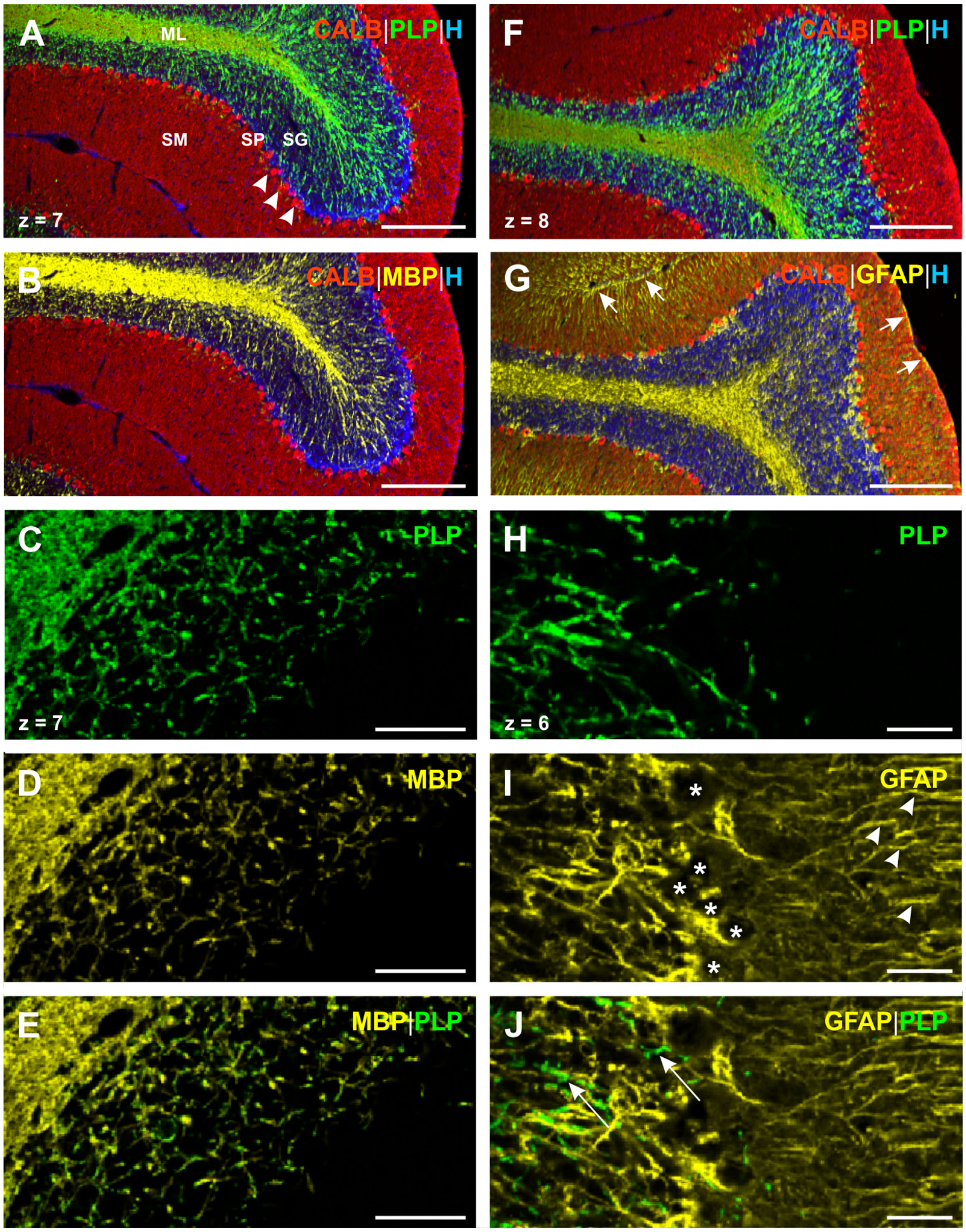

Supplementary Figure S1. Control stainings for PLP, MBP and GFAP in the murine cerebellum. **A-E**: Quadruple staining for CALB, PLP, MBP and Hoechst (N° ①, Table 1) shows a complete co-localization for MBP and PLP. These two myelin proteins are mostly present in the medullary layer and the stratum granulare. CALB+ -Purkinje cells define the stratum purkinjense of the cerebellum (**A**, arrowheads). **F-J**: Quadruple staining for CALB, PLP, GFAP and Hoechst (N° ②, Table 1) shows a different distribution of PLP and GFAP: While PLP+ -fibers end in the lower stratum moleculare, GFAP can be detected in all cerebellar layers. GFAP+ -Bergmann glial cells surround the pyknic cell bodies of the Purkinje cells (**I**, asterisks) and afterwards dispatch their radial aligned processes (**I**, exemplarily indicated by arrowheads) forming the membrana limitans gliae superficialis at the cerebellar surface (**G**, short arrows). GFAP and PLP showed no co-localization (**J**, long arrows). CALB: Calbindin D28k; GFAP: Glial fibrillary acidic protein; H: Hoechst; MBP: Myelin basic protein;

ML: Medullary layer; PLP: Proteolipid protein; SG: Stratum granulare; SM: Stratum moleculare; SP: Stratum purkinjense. Z-step = 1 µm; scale bars 200 µm (A,B,F,G), 50 µm (C–E), 25 µm (H-J).

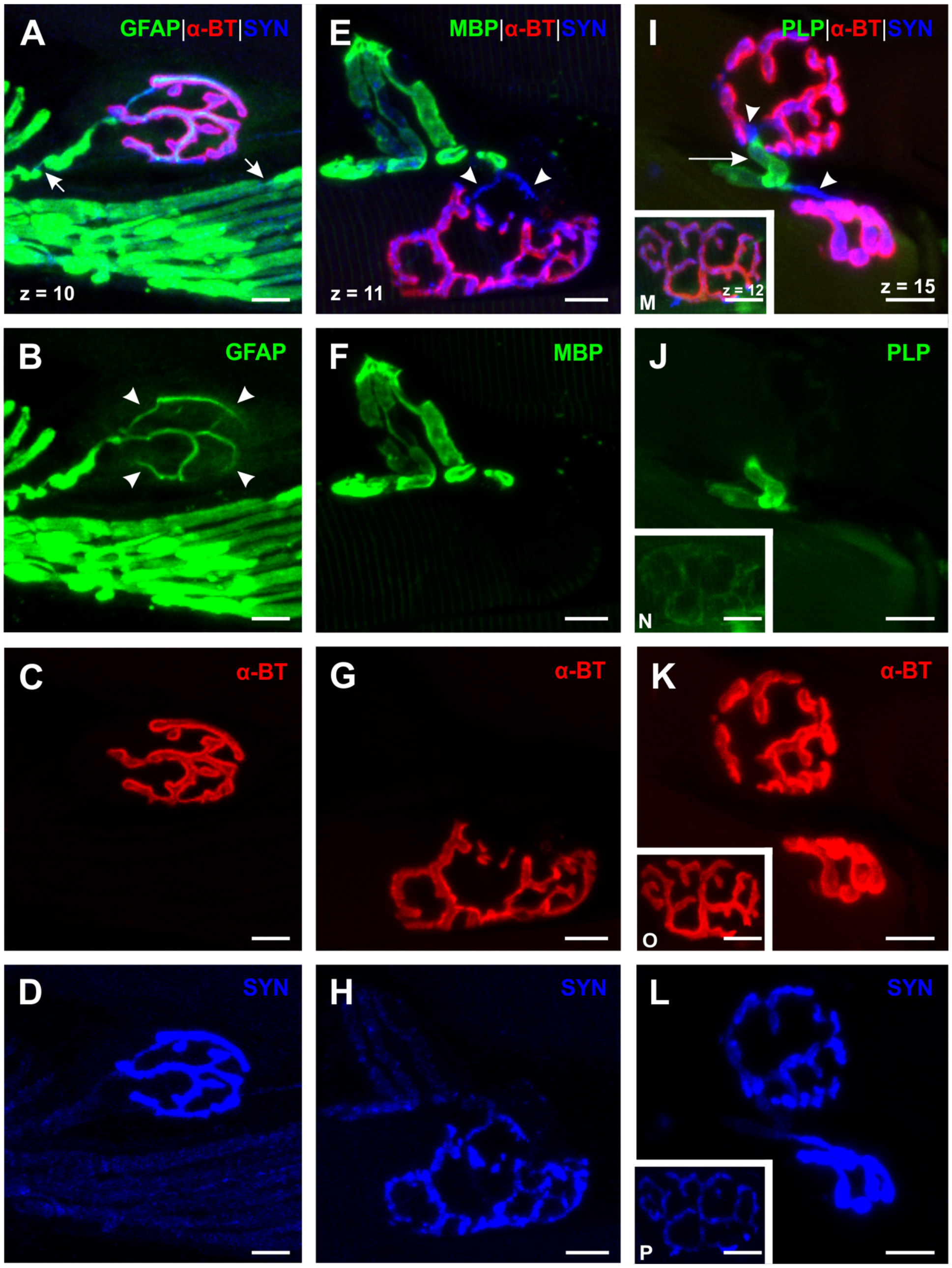

Supplementary Figure S2. Expression of GFAP, MBP and PLP in the NMJ of the tibialis anterior muscle. **A-D**: Triple staining for GFAP, α-BT and SYN (N° ③, Table 1) indicates the presence of GFAP as a cytoskeletal component of peripheral myelinating Schwann cells. GFAP⁺-PSCs accompany the axons after myelin loss resulting in a framework like structure (**B**, arrowheads). In the peripheral nerve bundle nodes of Ranvier can be found (**A**, short arrows). **E-H:** Triple staining for MBP, α-BT and SYN (N° ④, Table 1) shows that MBP expression is restricted to the myelin sheaths of the SYN⁺-motor axons (**F**). Prior to its associated motor endplate, the myelinated motor axon loses the myelin sheath and from there on only the SYN⁺-axon can be found (**E**, arrowheads). MBP was not detectable at the protein level in PSCs. **I-P:** Quadruple staining of PLP, α-BT, SYN and Hoechst (Hoechst not shown; N° ⑤, Table 1). PLP showed the same distribution in the myelin sheath of endplate-related efferences as MBP (**I**, arrow and arrowheads). In contrast, in some cases PLP was detected in the neuromuscular junction, forming a PLP⁺-relief of the endplates (**N**), while in other cases PLP could not be found (**J**). α-BT: α-Bungarotoxin; GFAP: Glial fibrillary acidic protein; MBP: Myelin basic protein; PLP: Proteolipid protein; SYN: Synaptophysin. Z-step = 1 μm; scale bars 10 μm.

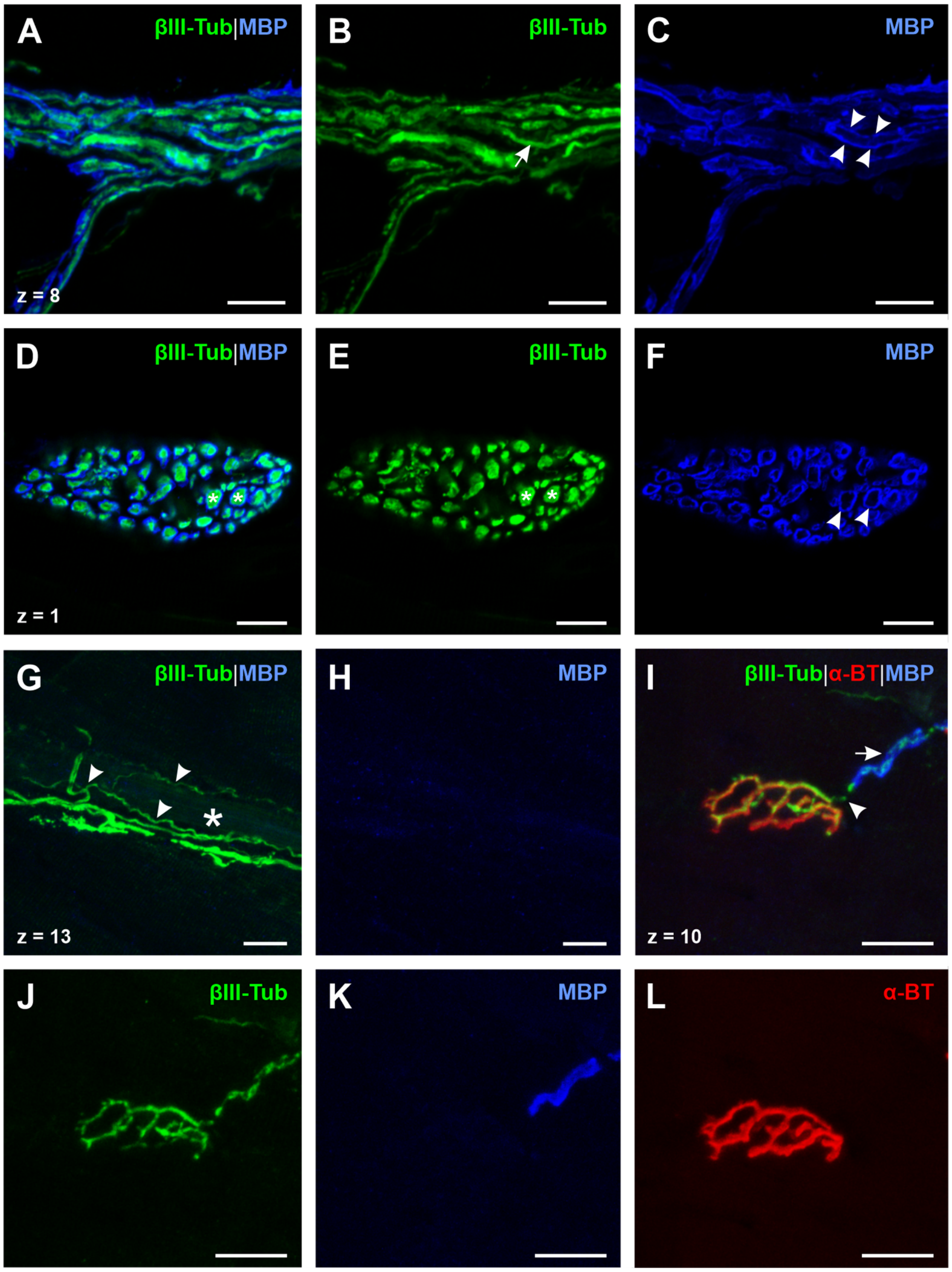

Supplementary Figure S3. Expression of βIII-tubulin and MBP in nerve fiber bundles, around blood vesels and in the NMJ of the tibialis anterior muscle. **A-H**: Triple staining of βIII-tubulin, MBP and α-BT (α-BT not shown, N° ⑧, Table 1). The longitudinal section (**A-C**) and the cross-section (**D-F**) of an intermuscular nerve fiber bundle allows the differentiation between axons (**B**, short arrow; **D-E**, asterisks) and the corresponding myelin sheath of the nerve fibers (**C** and **F**, arrowheads). **G-H**: Gracile blood vessel-related nerve fibers, wrapped around the outer vessel wall, appear to be βIII-tubulin-positive (**G**, arrowheads) but show no positiveness for MBP (**H**), thus proving not to be myelinated. The lumen is marked by the asterisk (**G**). **I-L**: The triple staining of βIII-tubulin, MBP and α-BT (N° ⑧, Table 1) in the NMJ confirms the staining results of SYN and ChAT (cf. Supplementary Figure S2 and S4), showing the loss of myelin of the myelinated motor axon (**I**, short arrow) close to its associated endplate. From there on the unmyelinated part of the βIII-tubulin$^+$ axon (**I**, arrowhead) continues to the presynaptic region of the motor endplate. α-BT: α-Bungarotoxin; ChAT: Choline acetyltransferase; MBP: Myelin basic protein; SYN: Synaptophysin. Z-step = 1 µm; scale bars 20 µm.

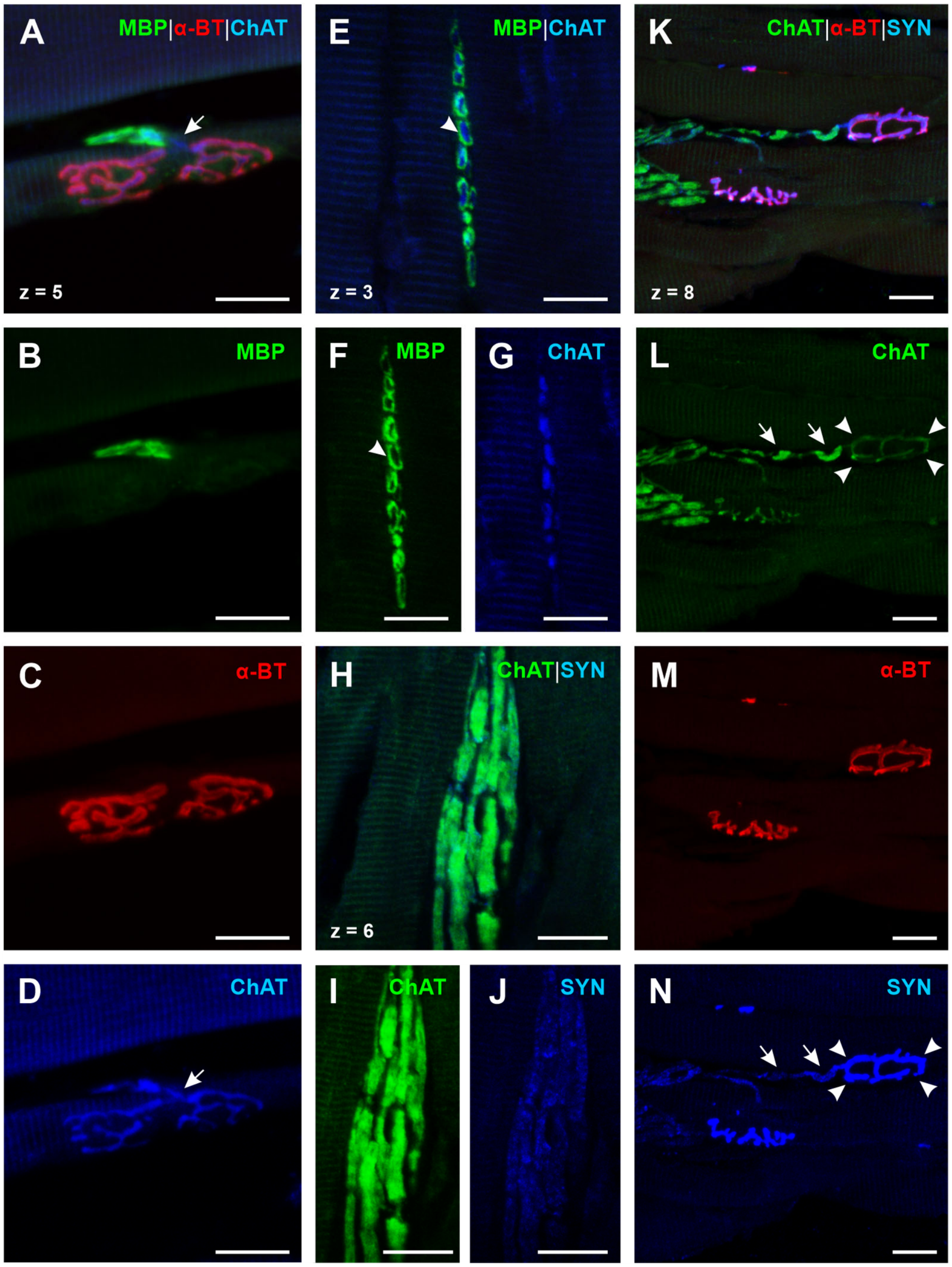

Supplementary Figure S4. Positive control stainings of ChAT and comparison of ChAT and synaptophysin distribution in the tibialis anterior muscle. **A-D**: Triple staining for ChAT, MBP and α-BT (N° ⑥, Table 1) indicates that MBP expression is restricted to the myelin sheaths of myelinated axons. In this triple staining, the common neuronal and axonal marker ChAT was used to verify the results of the SYN staining shown in Supplementary Figure S2. Prior to its associated motor endplate, myelin loss of the motor axon can be detected. From there on, only the ChAT^{+}-axon can be found (**A** and **D**, short arrows). **E-G**: Triple staining for ChAT, MBP and α-BT (N° ⑥, Table 1) shows nerve fiber bundles embedded between muscle fibers. The axonal cross-sections allow the differentiation of myelin sheaths (**E** and **F**, exemplarily indicated by arrowhead) and enwrapped axons, marked by ChAT (**G**). **H-N**: Triple staining for ChAT, SYN and α-BT (N° ⑦, Table 1) proves the colocalization of ChAT and SYN in nerve fibers, even though SYN staining is more slightly present (**H-J**). This staining result is explainable since SYN is a common presynaptic marker, thus accumulating in the end range of the motor axon (**N**, arrowheads). Moreover, a decreasing signal is detectable with increasing distance to the endplate (**N**, short arrows). However, ChAT distribution is opposite to

SYN, showing only a faint staining in the endplate region (**L**, arrow heads) and an increasing signal along the axon (**L**, short arrows). These results underline the role of SYN as a presynaptic marker of the endplate region, whereas ChAT is a more suitable marker for efferent axons. α-BT: α-Bungarotoxin; ChAT: Choline acetyltransferase; MBP: Myelin basic protein; SYN: Synaptophysin. Z-step = 1 μm; scale bars 10 μm (E-G), 20 μm (A-D and H-N).
